# Optimization of the 4-anilinoquin(az)oline scaffold as epidermal growth factor receptor (EGFR) inhibitors for chordoma utilizing a toxicology profiling assay platform

**DOI:** 10.1038/s41598-022-15552-5

**Published:** 2022-07-27

**Authors:** Andrew A. Bieberich, Tuomo Laitinen, Kaitlyn Maffuid, Raymond O. Fatig, Chad D. Torrice, David C. Morris, Daniel J. Crona, Christopher R. M. Asquith

**Affiliations:** 1 AsedaSciences Inc., 1281 Win Hentschel Boulevard, West Lafayette, IN 47906 USA; 2 School of Pharmacy, Faculty of Health Sciences, University of Eastern Finland, 70211 Kuopio, Finland; 3 Division of Pharmacotherapy and Experimental Therapeutics, UNC Eshelman School of Pharmacy, University of North Carolina at Chapel Hill, Chapel Hill, NC 27599 USA; 4 UNC Catalyst for Rare Diseases, UNC Eshelman School of Pharmacy, University of North Carolina at Chapel Hill, Chapel Hill, NC 27599 USA; 5 Lineberger Comprehensive Cancer Center, University of North Carolina at Chapel Hill, Chapel Hill, NC 27599 USA; 6 Structural Genomics Consortium and Division of Chemical Biology and Medicinal Chemistry, UNC Eshelman School of Pharmacy, University of North Carolina at Chapel Hill, Chapel Hill, NC 27599 USA; 7 Department of Pharmacology, School of Medicine, University of North Carolina at Chapel Hill, Chapel Hill, NC 27599 USA

**Keywords:** Bone cancer, Cancer models, Cancer, Biological techniques, Cancer, Cell biology, Chemical biology, Chemical libraries, Computational chemistry, Lead optimization, Pharmacology, Screening, Structure-based drug design, Target identification, Target validation, Drug discovery, Medicinal chemistry, Target identification, Target validation, Chemistry, Chemical biology, Medicinal chemistry, Organic chemistry, Chemical synthesis

## Abstract

The 4-anilinoquin(az)oline is a well-known kinase inhibitor scaffold incorporated in clinical inhibitors including gefitinib, erlotinib, afatinib, and lapatinib, all of which have previously demonstrated activity against chordoma cell lines in vitro. We screened a focused array of compounds based on the 4-anilinoquin(az)oline scaffold against both U-CH1 and the epidermal growth factor receptor (EGFR) inhibitor resistant U-CH2. To prioritize the hit compounds for further development, we screened the compound set in a multiparameter cell health toxicity assay. The de-risked compounds were then screened against a wider panel of patient derived cell lines and demonstrated low micromolar efficacy in cells. We also investigated the properties that gave rise to the toxophore markers, including the structural and electronic features, while optimizing for EGFR in-cell target engagement. These de-risked leads present a potential new therapeutic avenue for treatment of chordomas and new chemical tools and probe compound **45** (UNC-CA359) to interrogate EGFR mediated disease phenotypes.

## Introduction

Cancer is the leading cause of death worldwide, responsible for nearly 10 million deaths in the last year^1,2^. Protein kinases have presented promising drug targets, with more than 70 inhibitors targeting the ATP binding site of kinases approved for use in the clinic^3^. This promising treatment avenue is currently being explored to treat chordomas using afatinib^4^.

Chordoma is an osseous cancer, contained within the spine and central nervous system, with invasive and metastatic potential which is driven by the notochord transcription factor brachyury^5^. Treatment of chordoma is challenging, with initial treatment focused on radical resection combined with proton beam therapy. Surgical and chemotherapeutic options are limited, meaning recurrent or metastatic disease is often fatal^5,6^.

**Figure 1:**
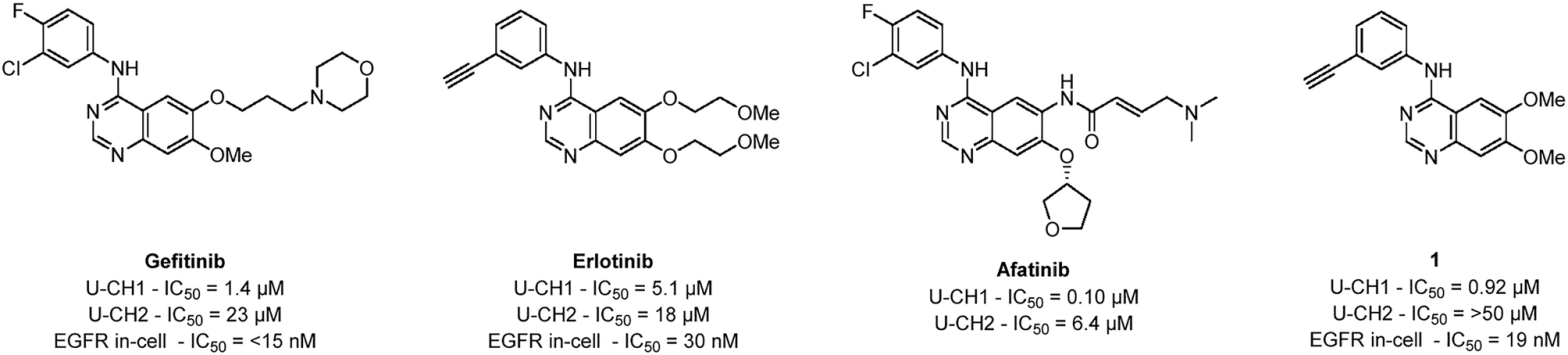
Previously reported inhibitors of chordoma and EGFR.

Epidermal growth factor receptor (EGFR) is highly expressed in 40% of chordomas, and targeting EGFR has been shown to be both effective in chordoma cell lines and murine xenograft models^4,7,8^. Several quinazoline-based clinical kinase inhibitors whose main target is EGFR have been shown to inhibit chordoma cell lines, including gefitinib, erlotinib, afatinib and lapatinib (Fig. 1)^4,8–12^.

While these drugs were designed to target EGFR, they show similar or higher affinity for several other kinases, making them limited tools for direct interrogation of EGFR and other kinase biology^12–17^. They are also not effective at targeting some of the more resistant patient derived cell lines^9,10,12^. While exploring cyclin G associated kinase (GAK) as a potential collateral target to treat chordoma, we identified a series of potent EGFR inhibitor starting points based on the 4-anilinoquinazoline scaffold, exemplified by **1**^10,12^.

## Results

**Scheme 1:**
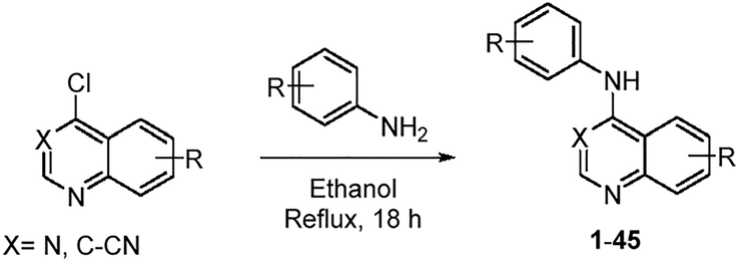
General synthetic procedure.

Our objectives are to simultaneously improve the chordoma cellular potency and compound preclinical safety profile. Our strategy was to interrogate the structure activity relationships (SAR) of these literature starting points, using our in-house GAK inhibitor knowledge and library, along with a safety risk estimation screen, to optimize the 4-anilinoquin(az)oline scaffold^18–23^. We initially synthesized and tested a panel of compounds based around compound **1**. We synthesized compounds **1**–**45** through nucleophilic aromatic displacement of 4-chloroquin(az)olines (Scheme 1) to furnish the products in good overall yields (20–97%) in most cases. Several lower yielding analogues included **10** (23%) and **16** (21%) both containing the trifluoromethyl quinazoline, and the 6,7-difluoroquinazoline analogue **6** (20%), all of which were consistent with previous reports^18,22^.

We set about optimizing the cellular potency of compound **1** with a series of  modifications, on both U-CH1 and U-CH2 chordoma cell lines. *N*-(3-ethynylphenyl)-6,7-dimethoxyquinazolin-4-amine (**1**) was previously identified as a narrow spectrum inhibitor of EGFR with only two other off-targets (GAK and Receptor-interacting serine/threonine-protein kinase 2 (RIPK2)) in a kinome wide screen^18^. Compound **1** was shown to be a low micromolar inhibitor of the U-CH1 cell line, consistent with our previous screen (Table 1)^10,12^. The corresponding 6-methoxy **2** had a slight drop in U-CH1 activity, while the unsubstituted analog **3** showed a substantial 25-fold drop. The introduction of a 6-methyl **4** restored half the lost inhibition on U-CH1 but removed all inhibition on U-CH2. Switching from a methyl to a fluorine **5** was equipotent; however, the 6,7-difluoro **6** doubled potency against U-CH1 back to IC_50_ = 6.8 μM while having no effect on U-CH2. The removal of the 7-fluoro and increasing of the size of the halogen from chloro to iodo **7**–**9** increased potency from IC_50_ = 25 μM to IC_50_ = 8.7 μM against U-CH1. The 6-trifluoromethyl **10** showed no improvement against U-CH1 but restored sub-100 µM activity against U-CH2 at IC_50_ = 42 μM.

**Table 1:** Initial screening of quinazolines against chordoma cell lines.


Name	R^1^	R^2^	U-CH1	U-CH2
			IC_50_ (μM)^a^	
**1**	OMe	OMe	0.63	66
**2**	OMe	H	1.4	47
**3**	H	H	35	52
**4**	Me	H	15	> 100
**5**	F	H	19	> 100
**6**	F	F	6.8	> 100
**7**	Cl	H	25	> 100
**8**	Br	H	16	> 100
**9**	I	H	8.7	> 100
**10**	CF_3_	H	21	42
**11**	H	OMe	1.9	1.2
**12**	H	F	41	> 100
**13**	H	Cl	21	> 100
**14**	H	Br	54	> 100
**15**	H	I	17	> 100
**16**	H	CF_3_	12	15
**17**	H	CN	65	> 100
**18**	CN	H	74	> 100
**19**	SO_2_Me	H	45	52
**20**	OCH_2_O		22	> 100
**21**	OCH_2_CH_2_O		8.8	> 100
**Erlotinib**	6,7-(OCH_2_CH_2_OMe)_2_		5.1	18

^a^Cell viability assay (n = 4).

Switching the 7-position back to the methoxy **11** yielded a compound with a potent activity profile against both cell lines, for the first time, with an IC_50_ = < 2 μM in both cases. Switching to a halogen at the 7-position, from fluorine to iodine (**12**–**15**), significantly reduced activity against U-CH1 compared with **11**, with no inhibition activity at all (IC_50_ = > 100 μM) on U-CH2. The 7-trifluoromethyl **16** showed some improvement against U-CH1 with an IC_50_ = 12 μM and some inhibition of U-CH2 (IC_50_ = 15 μM). The 6-cyano **17** and 7-cyano **18** substitutions demonstrated a sixfold reduction in inhibition of U-CH1 compared with **16**. The 6-methylsulfone **19** showed little improvement. However, reverting to an electron donating substituent cyclizing the catechol first with a methyl spacer **20** and then with an ethyl spacer **21** moved potency back toward single digit micromolar IC_50_ values against U-CH1. Compound **21** had a similar inhibition profile to erlotinib on U-CH1, but while erlotinib showed activity against U-CH2 (IC_50_ = 18 μM), **20** and **21** did not (IC_50_ = > 100 μM).

We then switched to the 3-cyanoquinoline scaffold, to evaluate if it was possible to increase the potency on U-CH1 and U-CH2, as had been demonstrated previously^10^. The initial compound 4-((3-ethynylphenyl)amino)-6,7-dimethoxyquinoline-3-carbonitrile (**22**) with the 6,7-dimethoxy substitution showed good inhibition against U-CH1 with an IC_50_ = 4.1 μM, and a weaker inhibition of U-CH2 (Table 2). Removal of the 7-methoxy to yield compound **23** afforded a compound with a fivefold reduction in U-CH1 inhibition and no observed effect on U-CH2. The unsubstituted analogue **24** showed a slight reduction in potency against U-CH1 compared with **23**. Smaller 6-position halogen substitutions were less favorable; as the size of the halogen increased from fluoro to iodo (**25**–**28**), so did the potency of the compound in both U-CH1 and U-CH2 cell lines. The 6-iodo substitution **28** was the most potent with U-CH1 IC_50_ = 8.7 μM and U-CH2 IC_50_ = 14 μM. The 6-methylsulfone **29** had only limited potency against both cell lines, while the 7-position halogens (**30**–**32**) showed no affect at all on either cell line. However, switching back to the 7-methoxy **33** recovered activity to levels observed in **22**.

**Table 2:** Initial screening of 3-cyanoquinolines against chordoma cell lines.


Name	R^1^	R^2^	U-CH1	U-CH2
			IC_50_ (μM)^a^	
**22**	OMe	OMe	4.1	36
**23**	OMe	H	20	> 100
**24**	H	H	34	> 100
**25**	F	H	23	> 100
**26**	Cl	H	14	40
**27**	Br	H	22	30
**28**	I	H	8.7	14
**29**	SO_2_Me	H	92	52
**30**	H	Cl	> 100	> 100
**31**	H	Br	> 100	> 100
**32**	H	I	> 100	> 100
**33**	H	OMe	4.1	40

^a^Cell viability assay (n = 4).

We then selected a subset of the most potent compounds on U-CH1 and U-CH2, along with some structurally diverse compounds (Table 3), to screen on four additional patient-derived chordoma cell lines (CH22, UM-Chor1, U-CH12, and U-CH7) and a human fibroblast cell line as a toxicity control (WS1)^10,12,24^. In addition, we ran an EGFR in-cell target engagement assay, measuring in-cell EGFR phosphorylation, using the human epidermoid carcinoma cell line A431. This cell line endogenously expresses a high level of EGFR, and stimulation of these cells with human epidermal growth factor (EGF) results in receptor tyrosine autophosphorylation^25^.

**Table 3:** Investigation of screening hits across four additional patient-derived chordoma cell lines and WS1 control.


Name	X	R^1^	R^2^	EGFR^b^	CH22	UM-Chor1	U-CH12	U-CH7	WS1
				IC_50_ (μM)^a^					
**6**	N	F	F	12	> 100	> 100	> 100	> 100	> 100
**9**	N	I	H	0.83	> 100	> 100	> 100	> 100	98
**11**	N	H	OMe	0.53	> 100	> 100	> 100	95	88
**15**	N	H	I	2.8	> 100	> 100	> 100	> 100	> 100
**16**	N	H	CF_3_	> 20	8.0	31	7.5	63	18
**17**	N	H	CN	> 20	> 100	> 100	> 100	> 100	> 100
**21**	N	OCH_2_CH_2_O		1.9	> 100	64	> 100	> 100	> 100
**Erlotinib**	N	6,7-(OCH_2_CH_2_OMe)_2_		0.30	> 100	19	68	95	19
**28**	C–CN	I	H	2.5	8.4	6.9	7.7	> 100	34
**32**	C–CN	H	I	2.7	> 100	> 100	> 100	> 100	> 100

^a^Cell viability assay (n = 4).

^b^ProQinase in-cell EGFR assay (n = 1).

*N*-(3-ethynylphenyl)-6,7-difluoroquinazolin-4-amine (**6**) displayed weaker activity against EGFR in A431 cells (IC_50_ = 12 μM), and no potency in the four additional patient-derived chordoma cell lines or WS1 control (IC_50_ = > 100 μM). The 6-iodo **9** was more potent against EGFR (IC_50_ = 0.83 μM), but was still weak in the four additional chordoma cell lines (IC_50_ = > 100 μM) and WS1 (IC_50_ = 98 μM). Similar results were observed for 7-methoxy **11** and 7-iodo **15**. The 6-trifluoromethyl **16**, while showing limited EGFR activity (IC_50_ = > 20 μM), displayed moderate activity against all four patient-derived chordoma cell lines, but this appeared to be driven by non-specific toxicity (WS1, IC_50_ = 18 μM). The 7-cyano **17** was selected for structural diversity but was inactive at the top concentration in all chordoma cell line assays (IC_50_ = > 100 μM). The closing of the 6,7-dimethoxy to form a 6-member ring **21** showed some activity against EGFR (IC_50_ = 1.9 μM), but only weak activity on UM-Chor1 (IC_50_ = 64 μM). Erlotinib performed consistently as previously described^10,12^, with good potency against EGFR (IC_50_ = 0.030 μM), weak activity in four of the patient-derived chordoma cell lines, but also with similar non-specific toxicity observed with compound **16** (IC_50_ = 19 μM). Switching to the 3-cyanoquinoline scaffold, 4-((3-ethynylphenyl)amino)-6-iodoquinoline-3-carbonitrile (**28**) showed an improvement in potency in three of the four chordoma cell lines: CH22 (IC_50_ = 8.4 μM), UM-Chor1 (IC_50_ = 6.9 μM), and U-CH12 (IC_50_ = 7.7 μM), with only moderate non-specific toxicity in the WS1 control cells (IC_50_ = 34 μM) despite an eightfold reduction in EGFR activity compared to erlotinib. The observed potency could be related to a target other than EGFR, as the corresponding 7-iodo **32** has similar EGFR activity to **28**, but was not potent in the patient-derived chordoma lines or the WS1 control (IC_50_ = > 100 μM).

To prioritize further optimization, in addition to cellular potency, we utilized the AsedaSciences® SYSTEMETRIC® Cell Health Screen. This live cell flow cytometry (FC) screen was developed to assess human safety risk for candidate pharmaceutical small molecules at the early hit-to-lead stage of development^26^. The screen works by estimating the risk using a supervised machine learning (ML) classifier to assess a multiparametric cellular stress phenotype, produced by the test compound, relative to a training set of known compounds (Table S1). This screen has been designed for early pipeline risk estimation regardless of pharmaceutical class/disease type, so it uses HL60 as the reporter cell line. While HL60 is not a direct chordoma model, it exhibits characteristics of practical importance for an automated flow cytometry platform assessing cell stress: suspension culture and optimal dynamic range with respect to the required physiological reporting dyes^26^.

The ML classifier was trained with 300 historically known compounds (on market or withdrawn drugs, research compounds, etc.), which were divided into two outcome classes based upon literature, clinical trials, and market histories where applicable^26^. This external curation informed an outcome of “high-risk” or “low-risk”. All 300 compounds were also processed through the automated FC screen, populating each of the two classes with frequency distributions of empirical cell-based phenotypes.

To estimate human safety risk for an unknown compound, the ML classifier assesses the similarity of the test compound’s cell-based phenotype to the distributions of phenotypes in the two outcome classes. The final quantitative score, or Cell Health Index (CHI), is the probability (0–1) that the test compound’s phenotype belongs in the distribution observed for the high-risk outcome class in the training set (Table 4). Phenotypes are derived from twelve FC detection parameters, including light scatter and a series of fluorescent physiological reporter dyes, that track cell stress-related endpoints.

**Table 4:**
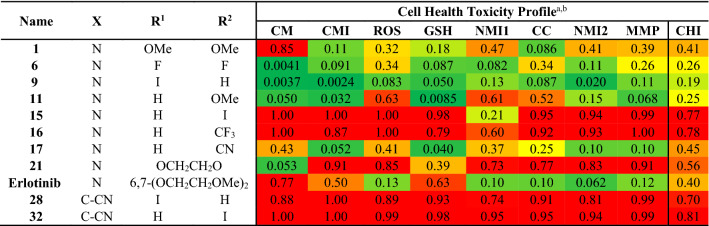
Toxicity profiling of the initial screening hits.

^a^Abbreviations from columns left to right. CM: Cell morphology; CMI: Cell membrane integrity; ROS: Reactive superoxide species; GSH: Glutathione; NMI1: Nuclear membrane integrity 1; CC: Cell Cycle; NMI2: Nuclear membrane integrity 2; MMP: Mitochondrial membrane depolarization; CHI: Cell Health Index.

^b^All n = 2.

To produce the Cell Health Index, the ML classifier uses all twelve FC parameters simultaneously in a multidimensional logistic regression model, so the CHI contains the most predictive power. In addition to the Cell Health Index, Table 4 also reports probability scores for eight cell stress-related endpoints. These scores are produced individually by passing information to the ML classifier relating to parameters that inform that endpoint. For example, to produce the cell morphology (CM) score, the ML classifier only uses the FC detection parameters for forward scatter (informing change in cell diameter) and side scatter (informing change in optical granularity of the cell) to inform the model. Together, these eight endpoints provide a “biological fingerprint”, allowing assessment of the degree to which SAR series members behave differently across endpoints.

The starting point *N*-(3-ethynylphenyl)-6,7-dimethoxyquinazolin-4-amine (**1**) produced an intermediate risk estimate, consistent with the WS1 toxicity counter screening^12^. The toxicity is potentially driven by cell morphology changes, but this is currently unclear. Compounds **6**, **9**, **11**, **17** and erlotinib were consistent and found to be non-toxic in both assays; in contrast liabilities were consistently found in **16** and **21**. The 7-iodo derivatives **15**, **28** and **32** all performed poorly in the cell health secondary screening but showed no toxicity in the initial WS1 (IC_50_ = > 100 μM) counter screen. Interestingly, the 6-iodo derivative **9**, appears to be more tolerated in this assay (for the complete data set see Tables S2–S3).

In order to optimize the scaffold further, the 6- and 7-positions on the quinazoline ring were fixed, and the aniline portion was modulated with different halogens (Table 5). The encouraging data across the panel of patient derived cell lines and toxicity profiling of the 6-iodo compound **9** meant the first set of analogues focused on small changes of the aniline on **9**. Shielding the anilino N–H group with a fluoro at the distal *ortho*-position **34**, maintained EGFR activity, but decreased U-CH1 inhibition by fourfold compared with **9**. Moving the fluoro to the proximal *ortho*-position **35** between the acetylene and quinazoline recovered the activity on U-CH1. The *para*-fluoro **36** was a threefold improvement in U-CH1 inhibition with EGFR activity maintained. Interestingly, switching to a *para*-chloro **37** reduced activity on U-CH1 by 11-fold, however, demonstrated relatively high activity on the difficult to treat U-CH2 cell line^9,10,12^, with an IC_50_ = 5.0 μM compared with no activity from the rest of the mini-series (**9**, **34**–**36**).

**Table 5:** Optimisation of **9**, **15** and **1** on EGFR and UCH-1 and UCH-2 chordoma cell lines.


Name	R^1^	R^2^	R^3^	R^4^	R^5^	U-CH1	U-CH2	EGFR^b^
						IC_50_ (μM)^a^		
**9**	I	H	H	H	H	8.7	> 100	0.83
**34**	I	H	F	H	H	27	> 100	0.76
**35**	I	H	H	F	H	8.9	> 100	1.9
**36**	I	H	H	H	F	2.7	> 100	2.7
**37**	I	H	H	H	Cl	31	5.0	2.0
**15**	H	I	H	H	H	17	> 100	2.8
**38**	H	I	F	H	H	15	> 100	5.2
**39**	H	I	H	F	H	2.4	15	1.7
**40**	H	I	H	H	F	> 100	> 100	3.6
**41**	H	I	H	H	Cl	9.1	16	3.7
**1**	OMe	OMe	H	H	H	0.63	66	0.019
**42**	OMe	OMe	F	H	H	6.7	20	0.29
**43**	OMe	OMe	H	F	H	2.0	7.1	0.060
**44**	OMe	OMe	H	H	F	> 100	22	0.030
**45**	OMe	OMe	H	H	Cl	> 100	35	0.018

^a^Cell viability assay (n = 4).

^b^ProQinase in-cell EGFR assay (n = 1).

The encouraging results with the 6-iodoquinazoline led to the exploration of the 7-iodo based around **15**. A fluoro at the distal *ortho*-position **38** showed no overall improvement, whereas in the proximal *ortho*-position **39** the fluoro improved potency on U-CH1 by sixfold (IC_50_ = 2.4 μM). Compound **39** also showed activity on the difficult to treat U-CH2 cell line (IC_50_ = 15 μM)^9,10,12^. The *para*-fluoro analog **40** showed no activity on U-CH1 or U-CH2, whereas the *para*-chloro derivative **41** demonstrated moderate inhibition on both U-CH1 (IC_50_ = 9.1 μM) and U-CH2 (IC_50_ = 16 μM) with low single digit micromolar EGFR inhibition (IC_50_ = 3.7 μM).

The 6,7-dimethoxy substitution present in **1** has demonstrated potent inhibition on U-CH1 below 1 µM, suggesting that this substitution pattern may yield a more potent compound across multiple cell lines. The distal *ortho*-fluoro **42** reduced activity on the U-CH1 by tenfold compared to **1**, whereas there was a threefold increase on U-CH2. This was despite **42** showing a tenfold decrease in in-cell EGFR activity. The proximal *ortho*-fluoro between the *meta*-acetylene and quinazoline ring system **43** demonstrated a threefold increase across U-CH1 and U-CH2 with EGFR activity increased by fivefold. The *para*-substituted analogues **44** and **45** both lost activity on U-CH1, but maintained some activity on U-CH2, and EGFR inhibition was maintained. The loss of inhibition on U-CH1 for **41**, **44** and **45** is not easily explained and highlights the degree to which target-ligand interactions in cells may be governed by energetic phenomena that classical models inadequately address^27^.

In order to better characterize these optimized compounds **34**–**45**, we screened them against the four additional patient-derived chordoma cell lines (CH22, UM-Chor1, U-CH12 and U-CH7) and WS1 as a nonspecific cytotoxicity control (Table 6). The 6-iodo analogues **34**–**37** demonstrated no inhibition against any of the four additional cell lines. The same was observed for the 7-iodo fluorinated analogues **38**–**40**, with the inhibition of **39** likely driven by nonspecific toxicity (WS1, IC_50_ = 40 μM). However, the 6-iodoquinazoline, *para*-chloro aniline analogue **41** showed some of the most potent inhibition to date on CH22 (IC_50_ = 0.48 μM) and U-CH12 (IC_50_ = 0.96 μM), along with good inhibition on the other two cell lines UM-Chor1 (IC_50_ = 25 μM) and U-CH7 (IC_50_ = 8.0 μM). There was some observed toxicity on WS1 (IC_50_ = 36 μM), but the ratios of inhibition to toxicity are 75-fold in the best case (CH-22/WS1). The 6,7-dimethoxy substitution analogues **42**–**45** showed good potential against the four cell lines but also showed some nonspecific toxicity in some cases. Compound **42** demonstrated good inhibition of CH22 (IC_50_ = 4.2 μM) but showed evidence of some non-specific toxicity (WS1, IC_50_ = 20 μM). The same was observed for **43**, with good inhibition of CH22 (IC_50_ = 5.8 μM) but some toxicity (WS1, IC_50_ = 12 μM). The *para*-fluoro analogue **44** demonstrated excellent potency, but this was unfortunately likely driven by nonspecific toxicity (WS1, IC_50_ = 0.33 μM). However, this nonspecific toxicity liability was able to be modulated with the *para*-chloro analogue **45** that showed potent inhibition on CH22 (IC_50_ = 1.2 μM) and U-CH12 (IC_50_ = 3.0 μM) with no observed toxicity (WS1, IC_50_ = > 100 μM).

**Table 6:** Optimized derivatives of **1**, **9** and **15** across four additional patient-derived chordoma cell lines and WS1 control.

Name	CH22	UM-Chor1	U-CH12	U-CH7	WS1
	IC_50_ (μM)^a^				
**9**	> 100	> 100	> 100	> 100	98
**34**	> 100	> 100	> 100	> 100	> 100
**35**	> 100	> 100	> 100	> 100	85
**36**	> 100	> 100	> 100	> 100	> 100
**37**	> 100	> 100	> 100	> 100	> 100
**15**	> 100	> 100	> 100	> 100	> 100
**38**	> 100	> 100	> 100	> 100	> 100
**39**	40	62	33	49	40
**40**	50	> 100	> 100	> 100	> 100
**41**	0.48	25	0.96	8.0	36
**1**	> 100	28	> 100	23	15
**42**	4.2	> 100	> 100	> 100	20
**43**	5.8	> 100	> 100	57	12
**44**	0.30	7.2	0.87	0.68	0.33
**45**	1.2	60	3.0	74	> 100

^a^cell viability assay (n = 4).

To further understand the toxicity liabilities in this scaffold we screened the optimized analogues **34**–**45** on the toxicity profiling platform and compared the results to some existing clinical compounds that have activity against chordoma (Table 7). *N*-(3-ethynylphenyl)-6-iodoquinazolin-4-amine (**9**) performed well in the toxicity profile platform, while a fluoro substitution in the distal *ortho*-position **34** and a *para*-position chloro **37** were not well tolerated. However, other substitutions, such as an *ortho*-position fluoro **35** and a *para*-position fluoro on the same core, were tolerated. Interestingly, moving the iodo from the 6-position **9** to the 7-postion **15** led to an increase in toxicity risk estimation in the assay. However, the modifications to the pendant aniline **38**–**41** were well tolerated, demonstrating that trends cannot always be easily defined. The original starting point **1** was also tolerated but near the middle of the Cell Health Index at 0.41. The modifications **42**–**45** were consistent with the profile of **1** showing an acceptable toxicity window consistent with the WS1 data. Erlotinib demonstrates low-to-intermediate risk for toxicity, but other relevant literature inhibitors show high toxicity risk, including gefitinib and tesevatinib^28–30^.

**Table 7:**
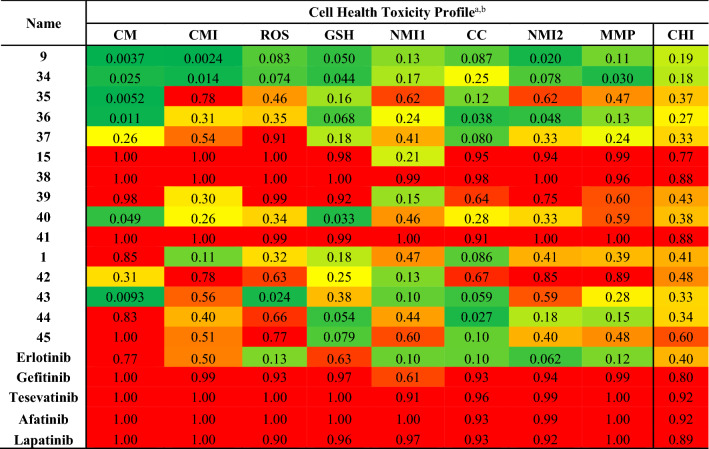
Optimization of **1**, **9** and **15** on EGFR and UCH-1 and UCH-2 chordoma cells lines.

^a^Abbreviations for columns from left to right. CM: Cell morphology; CMI: Cell membrane integrity; ROS: Reactive superoxide species; GSH: Glutathione; NMI1: Nuclear membrane integrity 1; CC: Cell Cycle; NMI2: Nuclear membrane integrity 2; MMP: Mitochondrial membrane depolarization; CHI: Cell Health Index. ^b^all n = 2.

**Figure 2:**
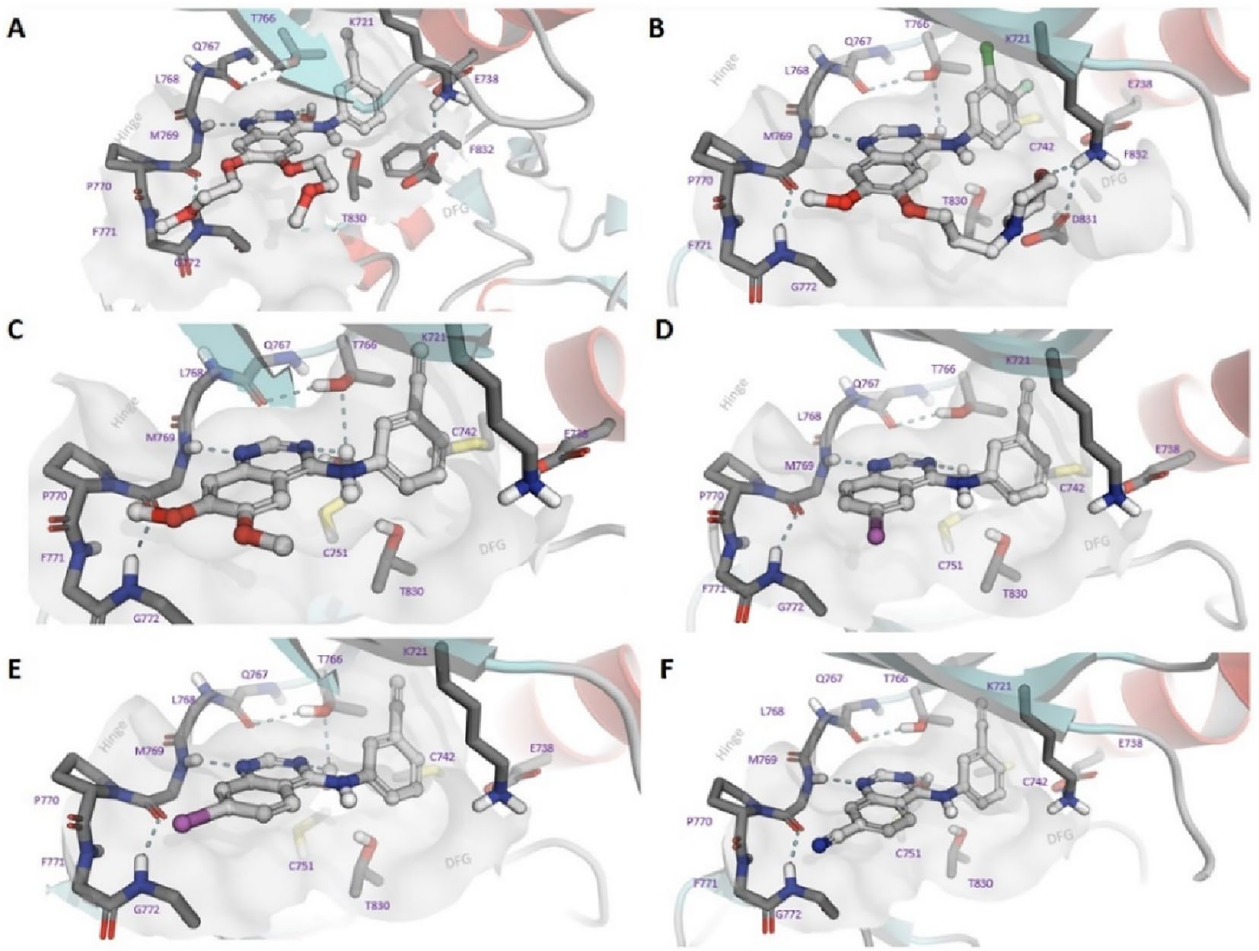
Docking of compounds in the EGFR ATP binding site (PDB:1M17): (**A**) erlotinib, (**B**) gefitinib, (**C**) **1**, (**D**) **9**, (**E**) **15**, (**F**) **17** showing key interactions with the hinge and hydrophobic pocket, along with the overall orientation of the quinazoline ring system.

**Figure 3:**
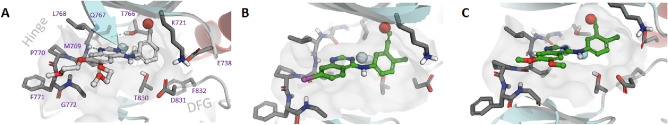
WaterMap simulation of Docking of compounds in the EGFR ATP binding site (PDB:1M17): (**A**) erlotinib, (**B**) **41** and (**C**) **45**. Red sphere shows locations of hight energy hydration site, which is replaced upon ligand binding. Light blue spheres behind the quinazoline scaffold are showing favourable hydration sites at back pocket are acting as bridge between quinazoline ring nitrogen and polar protein groups.

We modelled the progress of compounds in EGFR, using the full EGFR kinase domain (Fig. 2) to understand how the compounds orientate in the ATP binding site using erlotinib and gefitinib as a guide^12,31^. We noted that each compound, while having a similar binding mode in the active site, presented the quinazoline rings system at a slightly different angle. Interestingly, when the aniline substituent is the same, this appeared to be solely driven by the solvent exposed 6,7-position. The lead compounds **41** and **45** have the same binding mode as the previous analogues (Figure S1). The key difference is that they are better able to displace the high energy water present in the hydrophobic part of the ATP binding site (Fig. 3)^10,23^. We then did a series of one microsecond molecular dynamic simulations on **41** and **45**, using erlotinib as a control, and found stable binding that further supported our water network theory (Figures S2–S3).

To understand the selectivity profile across the kinome we screened **41** and **45** using radiometric protein kinase assays (^33^PanQinase® activity assay) to measure the kinase activity of the 320 protein kinases at two concentrations (10 μM and 1 μM) (Table S6). Compound **41** only inhibited EGFR at 1 μM (> 50%), while at 10 μM (> 50%), several other kinases in addition to EGFR were moderately inhibited including Aurora-B, ERBB4, MAP4K4, PRKG2 and SLK. Compound **45** had a similar profile with only EGFR potently inhibited at 1 μM (> 50%), with some moderate inhibition of ERBB2 and ERBB4. The higher concentration of 10 μM (> 50%) affected several additional kinases in addition to EGFR including Aurora-C, DDR2, EPHA6, EPHB2, LCK, MAP4K4, MAP4K5, MINK1, MKNK1, MKNK2, NIK, PIM1, RIPK2, SLK, and VEGFR2. Beyond the kinases that can be screened in this assay format, GAK^19,20^, NLK^19,20^, STK10 also known as LOK^22^ and ADCK3 also known as COQ8A^19,20,32^ could also be potentially inhibited by this scaffold.

## Discussion

Chordoma tumors are challenging to effectively treat. The integrated nature of the tumor, combined with slow growth, creates a uniquely complex situation where standard treatment options are not well suited. These factors, combined with highly variable presentation and susceptibility to treatment, prevent a readily available and generalizable therapeutic approach. There have been a number of clinical trials that have evaluated EGFR inhibitors to treat chordomas, but treatment outcomes are highly variable, and molecular mechanisms underlying this observed variability are not well understood^9,12^.

Increased EGFR inhibition generally increased the efficiency of inhibition on the chordoma cell lines. This further emphasizes the empirical observations in the literature and clinic. There are a number of reports that support EFGR's involvement in chordoma^9,10,12^. This was supported by the observation that **6**, **11**, **21**, **28** and erlotinib all had single digit micromolar inhibition on U-CH1 and good EGFR activity (Tables 1, 2, 3). However, when screening on a broader range of 5 cell lines, **6**, **11**, **21**, **28** each showed limited efficacy. This was the case despite both 6-iodo **9** and 7-methoxy **11** displaying very potent in-cell activity against EGFR (IC_50_ = 0.83 μM and 0.53 μM, respectively). However, in later optimized derivatives (**34**–**45**), more correspondence between EGFR and cell line inhibition was observed (Tables 5, 6). There were still exceptions, including **44** and lead compound **45**, both showing potent EGFR inhibition (IC_50_ = < 100 nM) but no activity against U-CH1 (IC_50_ = > 100 μM), again highlighting the complexity of chordoma biology. This was despite **45** showing exciting results across all other cell lines with limited toxicity on WS1 and potent in-cell activity against EGFR, with IC_50_ = 0.018 μM. U-CH2 proved difficult to inhibit with the most potent compound **11** already known^10^, and even the lead compounds presented here, **41** and **45,** only moderately inhibited U-CH2 (IC_50_ = 16 μM and 35 μM, respectively). Compounds **41** and **45** both have good inhibition across the remaining chordoma cell lines screened. This activity, combined with limited toxicity in WS1 (IC_50_ = 36 μM and > 100 μM, respectively), support from the flow cytometry toxicity assay and a very narrow kinome spectrum at 1 μM, are all desirable characteristics for a lead compound. Compounds **41** and **45** not only have potent inhibition of EGFR, and good in-cell target engagement (IC_50_ = 3.7 and 0.018 μM, respectively), they also appear to have stronger retention in the EGFR binding site than erlotinib via MD simulation (Figure S1).

The two lead compounds **41** and **45** are potent narrow spectrum EGFR inhibitors that provide exciting starting points for further optimization towards a potenital clinical compound to combat chordoma. Compound **45** (UNC-CA-359) is a probe quality compound for EGFR, fulfilling several of the following criteria: (1) in vitro biochemical IC_50_ = < 50 nM, (2) ≥ 30-fold selectivity relative to other kinases in a large assay panel, and (3) cellular activity or target engagement IC_50_ = < 1 μM^17^.

## Conclusion

This screening and optimization effort has produced a series of useful tools for further interrogation of EGFR biology, and more specifically, chordoma inhibitor development. The lead compounds **41** and **45** (UNC-CA359) both show very narrow kinome profiles and excellent activities across an array of patient derived chordoma cell lines. This is a potentially exciting step forward for a disease with a poor clinical prognosis.

## Experimental methods

### Biology and screening

#### Cell culture method

Chordoma cell lines were cultured as described previously^10,12,33,34^. Briefly, U-CH1 and U-CH2 cell lines were cultured in 4:1 IMDM:RPMI medium supplemented with 10% fetal bovine serum (FBS) and 1% penicillin/streptomycin in gel-coated flasks. The WS1 cell line (ATCC:CRL-1502) was cultured in DMEM medium supplemented with 10% FBS and 1% penicillin/streptomycin. CH22, UM-Chor1, U-CH12, and U-CH7 cell lines were cultured in RPMI medium supplemented with 10% FBS and 1% penicillin/streptomycin. The cell lines all undergo regular mycoplasma screening^9^.

#### Cell viability assays

Cells were seeded in 384 well plates and were treated with test compound in quadruplicate 24 h after plating. Cell viability was assessed at 72 h using alamarBlue (ThermoFisher, USA). Fluorescence was measured using a Tecan Infinite 200 PRO plate reader with excitation at 535 nm and emission at 590 nm. IC_50_ values were determined by four-parameter nonlinear regression analyses using GraphPad Prism™ version 8 software (San Diego, CA).

### Computational molecular modelling

Computational modelling was performed using Schrödinger Maestro software.

#### Protein preparation

Prior to docking studies, the selected x-ray structure of EGFR complexed with 4-anilinoquinazoline inhibitor erlotinib (PDB:1M17)^31^ was downloaded from RCSB, pre-processed and minimized using the protein preparation wizard tool of Schrödinger Suite 2020-4 (Protein Preparation Wizard uses modules: Epik; Impact and Prime, Schrödinger, LLC, New York, NY, 2020). Structures of small molecule ligands were parametrized and minimized using LigPrep module (LigPrep, Schrödinger, LLC, New York, NY, 2020) using OPLS3e force field^35^.

#### Molecular docking studies

Docking was computed using Induced Fit Docking workflow of Schrödinger employing SP-setting for Glide docking (Glide, Schrödinger, LLC, New York, NY, 2020) and side chains 5 Å from initiative ligand poses were consider for conformational refinement with Prime module. At both stages of docking hydrogen bond was required to hinge amine of Met769. Graphical illustrations are made using PyMOL Molecular Graphics System, Version 2.4.1. Schrödinger, LLC.

#### Hydration site analysis

Schrödinger suite 2020-4 WaterMap (WaterMap, Schrödinger, LLC, New York, NY, 2020) was used to evaluate the hydrations sites replaced upon ligand binding^23,36,37^. WaterMap links molecular dynamics (MD) simulations with statistical thermodynamic analysis of water molecules within a protein structure. Water molecules were analyzed within 6 Å from the docked ligand, and the 2 ns simulation was conducted with OPLS3e force field^35,38,39^.

#### Molecular dynamics

simulations were carried out using Schrödinger Desmond (Schrödinger Release 2020-2: Desmond Molecular Dynamics System, D. E. Shaw Research, Maestro-Desmond Interoperability Tools, Schrödinger, New York, NY, 2020). Selected favorable Induced fit docking poses of corresponding EGFR inhibitors were selected as template structures. Prior to simulation set up each kinase-inhibitor complex structure was first minimized using Protein preparation wizard of Schrodinger with default heavy atom rmsd constraint of 0.3 Å and OPLS3e force field. In Desmond system builder the orthorhombic periodic the system was created, and it was solvated using TIP3 waters and 0.1 M NaCl– salt buffer. At the beginning of the simulation, the system was subjected to default relaxation protocol of Desmond and heated up to simulation temperature of 300 K. Unconstrained 1 s simulations were run using NPT protocol at temperature of 300 K, pressure of 1.01325 bar, Noe–Hoover thermostat and timestep of 2 fs. Trajectories were visually examined to see whether docking poses are stable, and interactions remain along the 1 s. The numerical analyzes were calculated in assistance of simulation quality analysis and simulation event analysis tools.

### Flow cytometry toxicity profiling

AsedaSciences SYSTEMETRIC Cell Health Screen.

1. Physical execution summary

In a 384-well platform, HL60 cells were exposed to a 10-step, 3 × dilution series of each test compound (5 nM–100 µM) for 4 h at 37 °C with 5% CO_2_. Each dilution series was screened in duplicate, occupying a total of 20 wells, allowing 16 test compounds to be assayed per plate. Each row contained one positive and one negative control well, for a total of 16 replicate positive/negative control pairs on each assay plate. Compound formatting, cell deposition, and dye application were performed robotically so that final assay conditions comprised 100,000 cells in a 40 µL volume. After the 4 h compound exposure, cells were immediately stained with a panel of fluorescent dyes that report physiological signatures of both mitochondrial dysfunction and gross cell stress. Fluorescence and forward/side-scatter data were collected using automated FC with no gating. FC data are processed by an automated algorithm for producing quality control measures and ML classification of compound phenotypes.

2. HL60 cell culture production

HL60 cells were produced as suspension cultures in glass 850 cm^2^ roller bottles with vented caps, at 1 RPM, 5% CO_2_, and 37 °C. Culture medium was RPMI 1640 without glucose, supplemented with 10 mM galactose and 10% dialyzed heat-inactivated FBS (Atlanta Biologicals). Culture density was maintained at or below 1 × 10^6^ cells/mL. Standard protocol for the Cell Health Screen is that a new production lineage of HL60 cells is started each month, and a crossover screen is performed in which the old and new production lineages are compared by using a set of 16 reference compounds to produce a known set of stress phenotypes (see supporting information). In this way, variation of screen performance is minimized by producing all screening cell populations within a narrow range of passage numbers, each checked for consistency of phenotypic performance with reference compounds. This process was performed prior to using cells to produce data for this study.

3. Test compound format, cell exposure, and staining

All compound formatting, cell exposure, and staining with reporter dyes was performed in this study according to a standard protocol for the Cell Health Screen, which is described as follows. Compounds were formatted in groups of 16, with DMSO blanks loaded in unused screen positions for any smaller compound groups required to finish the complete study set. Each set of 16 test compounds was formatted in two replicate 384-well plates (Eppendorf Protein LoBind®, catalog number 951040589) for assays with two subsets of fluorescent dyes (Spectral overlap and DMSO limitation prevent simultaneous use of the complete dye panel in a single plate.). Compounds in these replicate plates were identical except for the positive controls, which were chosen to produce an optimal response within each subset of fluorescent reporter dyes. Test compound dilution series and controls were formatted on a Biomek® 4000. Each compound was formatted as a 10-step, 3 × dilution series, in duplicate, on each of the two plates. Negative control wells contained the diluent used for both the test compound dilution series and positive controls. The diluent was RPMI 1640 (supplemented as above) with final working concentration of DMSO normalized to 1% in all wells. The positive and negative controls were distributed to plate wells from a single initial reservoir of each control mixture. Final assay concentration range for test compounds was 5 nM–100 µM. Prior to cell deposition, assay plates containing formatted compounds were sealed and stored at room temperature, protected from light, for 2 h, to allow binding equilibrium between serum components and test compounds. A Biomek NX^P^ (Beckman Coulter) was used to deposit cells in all wells, at a density of 2.5 × 10^6^ cells/mL, in a final assay volume of 40 µL per well (approximately 100,000 cells per well). After cell deposition, each assay plate was sealed with breathable plate sealer, shaken at 2200 RPM for 10 s (Illumina® High-speed microplate shaker), and incubated for 4 h at 37 °C with 5% CO_2_.

- First fluorescent dye mix staining conditions

The following protocol was applied to the first plate in each assay plate pair, inclusive of all compounds in this study. Dye mix buffer was 1 × PBS with 4% FBS, filter sterilized. The dye set consisted of Calcein AM, SYTOX™ Red, MitoSOX™ Red, and Monobromobimane (Life Technologies catalog numbers C1430, S34859, M36008, and M20381, respectively). Dye concentrations were previously optimized, during the screen prototyping phase, to produce maximum dynamic range between positive and negative control wells. Prior to deposition of dye mix, the assay plate was removed from its 4-h incubation, and cells were gently pelleted at 300xg for 2 min. A Biomek NX^P^ (Beckman Coulter) was then used to aspirate 20 µL of each well volume, after which 20 µL of dye mix was deposited in all wells. After dye deposition, the plate was re-sealed with its breathable plate sealer, shaken 2 × at 2200 RPM for 5 s each time (1 s interval), and incubated for 10 min at 37 °C with 5% CO_2_. The plate was then rapidly cooled to room temperature for 1 min in a shallow water bath, after which acquisition of flow cytometry data was started immediately.

3b. Second fluorescent dye mix staining conditions

The following protocol was applied to the second plate in each assay plate pair, inclusive of all compounds in this study. Dye mix buffer was 1 × PBS with 4% FBS, filter sterilized. The dye set consisted of JC-9, propidium iodide, and Vybrant® DyeCycle™ Violet (Life Technologies catalog numbers D22421, P3566, and V35003, respectively). Dye concentrations were previously optimized, during the screen prototyping phase, to produce maximum dynamic range between positive and negative control wells. Cell pelleting and dye deposition were performed as above, in 3a. After dye deposition, the plate was re-sealed with its breathable plate sealer, shaken 2 × at 2200 RPM for 5 s each time (1 s interval), and incubated for 30 min at 37 °C with 5% CO_2_. The plate was then allowed to sit at room temperature for 15 min, protected from light. Acquisition of flow cytometry data was started immediately after this 15-min period.

4. Acquisition of flow cytometry data

FC data were acquired with a CyAn™ ADP flow cytometer (Beckman Coulter) with automated sampling performed by a HyperCyt® autosampler (Intellicyt). Autosampler settings were optimized to aspirate ≥ 10,000 cells per well. As described in section “Discussion”, the complete set of fluorescent dyes was applied as two non-overlapping mixtures on replicate assay plates. Therefore, two separate FC acquisition protocols with different sets of detection channels were used. Note that all channels were acquired with no gating. Triggering was on Forward Scatter with Threshold = 5%. Acquisition channel settings in Summit (version 4.3) for these two protocols are reported in Bieberich et al.^26^.

5. Data processing and analysis

All well-specific FC data and matching plate map files were transferred to an EC2 server instance on Amazon Web Services (AWS). An automated algorithm converts the raw data to risk scores for each compound in two stages:

- Feature reduction

For each test each compound, all 12 ungated FC detection parameters were converted to a feature vector as follows. For each of the 10 concentration steps in a test compound dilution series, quadratic form (QF) distance was calculated between the empirical distribution of an FC detection parameter and that same parameter in the negative control^26^. This effectively quantitates the amount of change, in test cells relative to the negative control cells, that each concentration of the test compound caused in one of the 12 FC detection channels. For each FC detection parameter, the amount of change between each test well and the negative control was thus converted to a dose–response curve of QF distance values. The same process was executed for all 12 FC detection parameters, after which each of the 12 QF distance value curves was further reduced to two values: the point of the maximum rate of change and the range within which change occurs^26^. These two feature values for each FC parameter were then assembled into a vector representing all 12 FC parameters. This vector serves as the quantitative digital phenotype for the test compound, to be used in subsequent ML classification^26^.

5b. Machine learning classification

Risk scores were produced for test compounds with an ML classifier employing supervised learning, with a multidimensional logistic model. The classifier was trained on a set of 300 known compounds drawn from on-market pharmaceuticals, withdrawn drugs, research compounds, and a few industrial/agricultural compounds (representative set shown in Table S1). First, all training set compounds were assigned to one of two binary outcome classes: the “yes” (expectation of high cell stress) or “no” class. This assignment was based upon manually curated external information from the scientific literature, clinical trial results, and commercial histories (where applicable). Each training compound was also screened to produce an empirical phenotypic feature vector, as described above. In this way, each of the two outcome classes in the training set was populated with an empirical distribution of cell-stress phenotypes from the FC screen. With these two data types attached to each training compound, historical outcome and empirical cellular phenotype, the goal of classifier training was to quantify the dependence of class membership on phenotype. This is a classic problem for an optimized logistic regression model. The classifier was trained by repeated cross-validation. Using the two training outcome classes, the logistic model optimization process sought the most parsimonious model allowing for maximum separation of the two populations of phenotypes. The optimally fit model then became the classification tool, allowing calculation of the probability that a feature vector, from any compound, could be assigned to the “yes” (high cell stress) class. Subsequently, for any test compound, the final risk score, or Cell Health Index (CHI), was the probability (maximum likelihood) with which the test compound's phenotypic feature vector could be assigned to the “yes” class defined by the training set. In addition, a series of lower-dimensional classifiers were trained on the same training set, calculating the probability of “yes” class assignment if only data for specific endpoints were considered. For example, the two FC detection parameters forward-scatter and side-scatter, from the 488 nm laser, were input to the classifier to produce the score called “cell morphology” (CM). These endpoint classifications produced a “biological fingerprint” of scores that can be interpreted as indicating relative contributions of each endpoint to the final multiparameter CHI score. However, note that the predictivity of the individual endpoints is not assumed to be equal, among themselves or to the CHI.

### Chemistry

#### General procedure for the synthesis of 4-anilinoquin(az)olines

4-Chloroquin(az)oline derivative (1.0 eq.), aniline (1.1 eq.) were suspended in ethanol (10 mL) and refluxed for 18 h. The crude mixture was purified by flash chromatography using EtOAc:hexane followed by 1–5% methanol in EtOAc. After solvent removal under reduced pressure, the product was obtained as a free following solid or recrystallized from ethanol/water. Compounds (**1**–**45**) were prepared as previously reported and were consistent with previous reports^22^.

## Supplementary Information

Supplementary Information.
