# Supplementary material for: Optimization of the 4-anilinoquin(az)oline scaffold as epidermal growth factor receptor (EGFR) inhibitors for chordoma utilizing a toxicology profiling assay platform
Source: Sci Rep. 2022 Jul 27;12:12820. doi: 10.1038/s41598-022-15552-5 (PMC9329436; doi:10.1038/s41598-022-15552-5)
Supplement: Supplementary file 1 — Supplementary Information. [file 41598_2022_15552_MOESM1_ESM.docx]

**Supporting Information**

**Optimization of the 4-anilinoquin(az)oline scaffold as epidermal growth factor receptor (EGFR) inhibitors for chordoma utilizing a toxicology profiling assay platform**

Andrew A. Bieberich, Tuomo Laitinen, Kaitlyn Maffuid, Raymond O. Fatig III, Chad D. Torrice, David C. Morris, Daniel J. Crona, Christopher R. M. Asquith*

^*^Email: christopher.asquith@uef.fi

**Contents**

**1. Toxicity Profiling Platform**

**2. Additional Modelling and Molecular Dynamic Simulations.**

**3. Kinome Scans of 41 and 45**

**4. In-Cell EGFR Dose Curves**

**5. Labbook Numbers and SMILES.**

**6. Mass Spectrometry Method.**

**1. Toxicity Profiling Platform**

**Table S1.** Representative sample of compound training set outcome classes for Cell Health Screen.

| **Name** | **Cell Health Toxcity Profile**^a,b^ | | | | | | | | |  |
| --- | --- | --- | --- | --- | --- | --- | --- | --- | --- | --- |
|  | **CM** | **CMI** | **ROS** | **GSH** | **NMI1** | **CC** | **NMI2** | **MMP** | **CHI** | **OC** |
| **Tamoxifen** | 1.00 | 1.00 | 1.00 | 1.00 | 1.00 | 0.98 | 0.99 | 1.00 | 0.94 | yes |
| **Terfenadine** | 1.00 | 1.00 | 1.00 | 1.00 | 0.99 | 0.98 | 0.99 | 1.00 | 0.92 | yes |
| **Prochlorperazine** | 1.00 | 1.00 | 1.00 | 1.00 | 1.00 | 0.96 | 0.99 | 1.00 | 0.9 | yes |
| **Thioridazine** | 1.00 | 1.00 | 1.00 | 1.00 | 1.00 | 0.89 | 1.00 | 1.00 | 0.85 | yes |
| **Berberine** | 0.99 | 0.050 | 1.00 | 0.93 | 0.99 | 0.99 | 0.97 | 1.00 | 0.82 | yes |
| **Nefazodone** | 0.97 | 1.00 | 1.00 | 0.99 | 0.73 | 0.97 | 0.87 | 1.00 | 0.78 | yes |
| **Promethazine** | 1.00 | 0.98 | 0.98 | 0.96 | 0.84 | 0.46 | 0.94 | 0.97 | 0.71 | yes |
| **Rotenone** | 0.93 | 0.83 | 0.94 | 0.79 | 0.54 | 0.70 | 0.76 | 0.99 | 0.66 | yes |
| **Amiodarone** | 0.97 | 0.80 | 0.19 | 0.71 | 0.36 | 0.96 | 0.55 | 0.87 | 0.60 | yes |
| **Celecoxib** | 0.56 | 0.94 | 0.96 | 0.36 | 0.53 | 0.61 | 0.51 | 0.51 | 0.54 | yes |
| **Loratadine** | 0.36 | 0.70 | 0.85 | 0.64 | 0.33 | 0.84 | 0.52 | 0.65 | 0.42 | no |
| **Phenformin** | 0.74 | 0.27 | 0.77 | 0.17 | 0.82 | 0.28 | 0.080 | 0.37 | 0.41 | no |
| **Rosiglitazone** | 0.49 | 0.23 | 0.070 | 0.53 | 0.23 | 0.27 | 0.27 | 0.050 | 0.37 | no |
| **Nilutamide** | 0.040 | 0.060 | 0.090 | 0.040 | 0.21 | 0.68 | 0.52 | 0.42 | 0.34 | no |
| **Tranilast** | 0.020 | 0.13 | 0.12 | 0.030 | 0.73 | 0.10 | 0.30 | 0.17 | 0.32 | no |
| **Phentolamine** | 0.00 | 0.16 | 0.11 | 0.17 | 0.64 | 0.040 | 0.79 | 0.29 | 0.30 | no |
| **Propranolol** | 0.060 | 0.20 | 0.18 | 0.11 | 0.69 | 0.24 | 0.23 | 0.010 | 0.27 | no |
| **Sulfasalazine** | 0.010 | 0.060 | 0.090 | 0.030 | 0.14 | 0.10 | 0.63 | 0.31 | 0.25 | no |
| **Simvastatin** | 0.030 | 0.080 | 0.11 | 0.41 | 0.29 | 0.12 | 0.10 | 0.060 | 0.24 | no |
| **Buspirone** | 0.060 | 0.040 | 0.05 | 0.080 | 0.070 | 0.11 | 0.040 | 0.020 | 0.22 | no |
| **Diphenhydramine** | 0.010 | 0.030 | 0.040 | 0.030 | 0.20 | 0.030 | 0.11 | 0.030 | 0.20 | no |
| **Zileuton** | 0.020 | 0.010 | 0.070 | 0.020 | 0.17 | 0.11 | 0.080 | 0.060 | 0.19 | no |
| **Nevirapine** | 0.020 | 0.050 | 0.28 | 0.030 | 0.020 | 0.070 | 0.040 | 0.010 | 0.18 | no |
| **Fexofenadine** | 0.00 | 0.040 | 0.16 | 0.030 | 0.22 | 0.020 | 0.010 | 0.020 | 0.18 | no |
| **Pirfenidone** | 0.00 | 0.090 | 0.020 | 0.040 | 0.040 | 0.060 | 0.030 | 0.010 | 0.17 | no |
| **Ribavirin** | 0.010 | 0.070 | 0.23 | 0.060 | 0.16 | 0.040 | 0.030 | 0.000 | 0.16 | no |
| **Telbivudine** | 0.010 | 0.040 | 0.030 | 0.070 | 0.11 | 0.020 | 0.050 | 0.010 | 0.15 | no |
| **Clonidine** | 0.00 | 0.030 | 0.040 | 0.10 | 0.15 | 0.060 | 0.040 | 0.010 | 0.14 | no |
| **Oxaprozin** | 0.00 | 0.090 | 0.16 | 0.030 | 0.040 | 0.060 | 0.12 | 0.000 | 0.14 | no |
| **Ketoprofen** | 0.00 | 0.030 | 0.18 | 0.010 | 0.11 | 0.050 | 0.010 | 0.010 | 0.13 | no |

The AsedaSciences SYSTEMETRIC Cell Health Screen uses a supervised machine learning classifier to estimate human safety risk for small molecule compounds. The classifier was trained on a set of 300 known compounds including on-market and withdrawn pharmaceuticals, research compounds, and a few agricultural/industrial compounds. All training compounds were curated using research literature and market/clinical histories, where applicable. Curation enabled assignment of each compound to an outcome class (OC, far right column). The “yes” class includes all compounds expected, based upon external information, to cause elevated cell stress phenotypes as a result of documented cytotoxicity mechanisms and/or poor human safety profiles. The “no” class includes compounds expected to cause little to no cell stress. All training compounds were then processed through the Cell Health Screen to produce flow cytometry-based phenotypes, as described in Methods. Class and phenotypic data were then used to optimize a multidimensional logistic regression model describing the dependence of class membership upon phenotype strength. For any unknown test compound, the trained classifier outputs the probability that the test compound phenotype belongs in the “yes” class, essentially quantifying the test compound’s phenotypic similarity to the known high safety risk compounds in the training set. This probability is the Cell Health Index. The 30 compounds listed above are a representative sample from the training set, for purposes of illustrating the types of compounds included and their phenotypic range. Column header key: CM=cell morphology, CMI=cell membrane integrity, ROS=reactive oxygen species (with some specificity for mitochondrial superoxide), GSH=glutathione, NMI1=nuclear membrane integrity 1, CC=cell cycle, NMI2= nuclear membrane integrity 2, MMP=mitochondrial membrane polarization, and CHI=Cell Health Index. For the eight endpoint scores other than CHI, the score is calculated by applying the classifier model *only* to the flow cytometry parameter(s) informing each endpoint.

**Table S2.** Complete Toxicity profiling of Table 1.

| **Name** | **R^1^** | **R^2^** | **Cell Health Toxcity Profile**^a,b^ | | | | | | | | | |
| --- | --- | --- | --- | --- | --- | --- | --- | --- | --- | --- | --- | --- |
|  |  |  | **CM** | **CMI** | **ROS** | **GSH** | **NMI1** | **CC** | **NMI2** | **MMP** | **CHI** |  |
| **1** | OMe | OMe | 0.85 | 0.11 | 0.32 | 0.18 | 0.47 | 0.086 | 0.41 | 0.39 | 0.41 |  |
| **2** | OMe | H | 0.24 | 0.62 | 0.17 | 0.038 | 0.42 | 0.38 | 0.068 | 0.019 | 0.31 |  |
| **3** | H | H | 0.96 | 0.99 | 0.98 | 0.98 | 0.53 | 0.86 | 0.76 | 1.00 | 0.74 |  |
| **4** | Me | H | 0.0051 | 0.21 | 0.20 | 0.12 | 0.059 | 0.65 | 0.17 | 0.49 | 0.31 |  |
| **5** | F | H | 0.0041 | 0.091 | 0.34 | 0.087 | 0.082 | 0.34 | 0.11 | 0.26 | 0.26 |  |
| **6** | F | F | 0.0084 | 0.0042 | 0.073 | 0.036 | 0.037 | 0.19 | 0.028 | 0.029 | 0.16 |  |
| **7** | Cl | H | 0.0047 | 0.030 | 0.20 | 0.011 | 0.13 | 0.10 | 0.053 | 0.015 | 0.16 |  |
| **8** | Br | H | 0.0037 | 0.0024 | 0.083 | 0.05 | 0.13 | 0.087 | 0.020 | 0.11 | 0.19 |  |
| **9** | I | H | 0.0063 | 0.38 | 0.088 | 0.029 | 0.069 | 0.071 | 0.054 | 0.013 | 0.21 |  |
| **10** | CF_3_ | H | 0.058 | 0.045 | 0.77 | 0.004 | 0.21 | 0.59 | 0.19 | 0.74 | 0.40 |  |
| **11** | H | OMe | 0.050 | 0.032 | 0.63 | 0.0085 | 0.61 | 0.52 | 0.15 | 0.068 | 0.25 |  |
| **12** | H | F | 0.058 | 0.045 | 0.77 | 0.004 | 0.21 | 0.59 | 0.19 | 0.74 | 0.40 |  |
| **13** | H | Cl | 0.045 | 0.079 | 0.52 | 0.010 | 0.2 | 0.13 | 0.018 | 0.25 | 0.33 |  |
| **14** | H | Br | 0.024 | 0.41 | 0.86 | 0.010 | 0.033 | 0.74 | 0.3 | 0.17 | 0.33 |  |
| **15** | H | I | 1.00 | 1.00 | 1.00 | 0.98 | 0.21 | 0.95 | 0.94 | 0.99 | 0.77 |  |
| **16** | H | CF_3_ | 1.00 | 0.87 | 1.00 | 0.79 | 0.6 | 0.92 | 0.93 | 1.00 | 0.78 |  |
| **17** | H | CN | 0.43 | 0.052 | 0.41 | 0.04 | 0.37 | 0.25 | 0.10 | 0.10 | 0.45 |  |
| **18** | CN | H | 0.0026 | 0.0079 | 0.042 | 0.023 | 0.33 | 0.27 | 0.094 | 0.11 | 0.18 |  |
| **19** | SO_2_Me | H | 0.0013 | 0.0057 | 0.055 | 0.044 | 0.36 | 0.33 | 0.089 | 0.21 | 0.22 |  |
| **20** | OCH_2_O | | 0.0019 | 0.0038 | 0.027 | 0.087 | 0.72 | 0.13 | 0.0045 | 0.016 | 0.18 |  |
| **21** | OCH_2_CH_2_O | | 0.053 | 0.91 | 0.85 | 0.39 | 0.73 | 0.77 | 0.83 | 0.91 | 0.56 |  |
| **Erlotinib** | 6,7-(OCH_2_CH_2_OMe)_2_ | | 0.73 | 0.74 | 0.0086 | 0.81 | 0.025 | 0.40 | 0.17 | 0.46 | 0.20 |  |

^a^CM: Cell morphology; CMI: Cell membrane integrity; ROS: Reactive superoxide species; GSH: Glutathione; NMI1: Nuclear membrane integrity 1; CC: DyeCycle Violet; NMI2: Nuclear membrane integrity 2; MMP: Mitochondrial membrane depolarization. ^b^assay n=2

**Table S3.** Complete Toxicity profiling of Table 2.

| **Name** | **R^1^** | **R^2^** | **Cell Health Toxcity Profile**^a,b^ | | | | | | | | |
| --- | --- | --- | --- | --- | --- | --- | --- | --- | --- | --- | --- |
|  |  |  | **CM** | **CMI** | **ROS** | **GSH** | **NMI1** | **CC** | **NMI2** | **MMP** | **CHI** |
| **22** | OMe | OMe | 0.35 | 0.77 | 0.81 | 0.071 | 0.67 | 0.33 | 0.29 | 0.39 | 0.40 |
| **23** | OMe | H | 0.020 | 0.0039 | 0.056 | 0.0085 | 0.30 | 0.74 | 0.11 | 0.28 | 0.26 |
| **24** | H | H | 0.99 | 1.00 | 0.98 | 0.98 | 0.85 | 0.76 | 0.81 | 0.97 | 0.70 |
| **25** | F | H | 0.98 | 0.99 | 0.93 | 0.97 | 0.71 | 0.76 | 0.72 | 0.96 | 0.69 |
| **26** | Cl | H | 1.00 | 1.00 | 1.00 | 0.99 | 0.98 | 0.93 | 0.97 | 0.99 | 0.83 |
| **27** | Br | H | 1.00 | 1.00 | 1.00 | 0.98 | 0.32 | 0.98 | 0.98 | 0.96 | 0.75 |
| **28** | I | H | 0.88 | 1.00 | 0.89 | 0.93 | 0.74 | 0.91 | 0.81 | 0.99 | 0.70 |
| **29** | SO_2_Me | H | 0.003 | 0.037 | 0.046 | 0.039 | 0.18 | 0.071 | 0.009 | 0.021 | 0.16 |
| **30** | H | Cl | 0.31 | 0.25 | 0.44 | 0.065 | 0.69 | 0.17 | 0.041 | 0.080 | 0.35 |
| **31** | H | Br | 0.95 | 0.88 | 0.87 | 0.92 | 0.45 | 0.92 | 0.34 | 0.84 | 0.59 |
| **32** | H | I | 1.00 | 1.00 | 0.99 | 0.98 | 0.95 | 0.95 | 0.94 | 0.99 | 0.81 |
| **33** | H | OMe | 0.97 | 0.90 | 0.68 | 0.78 | 0.20 | 0.81 | 0.16 | 0.22 | 0.44 |

^a^CM: Cell morphology; CMI: Cell membrane integrity; ROS: Reactive superoxide species; GSH: Glutathione; NMI1: Nuclear membrane integrity 1; CC: DyeCycle Violet; NMI2: Nuclear membrane integrity 2; MMP: Mitochondrial membrane depolarization. ^b^assay n=2

**2. Additional Modelling and Molecular Dynamic Simulations.**


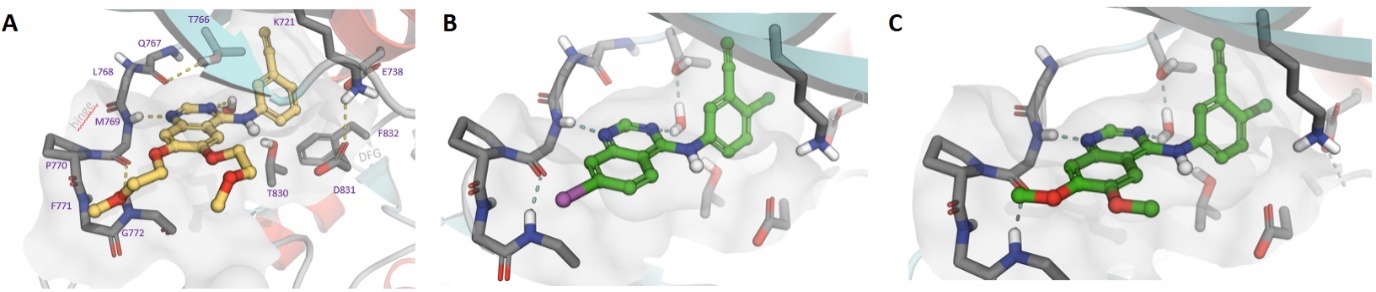


**Figure S1**. A detailed look at water positioning using docking: A) Erlotinib, B) **41**, C) **45**.


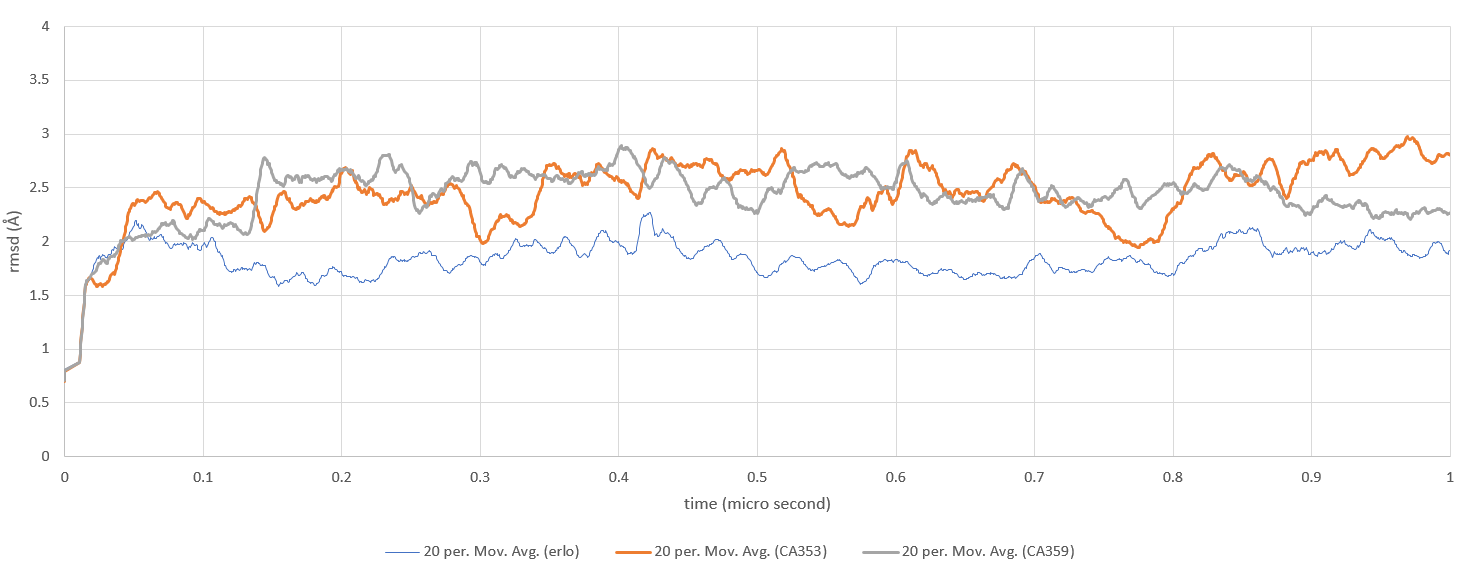


**Figure S2**. Molecular Dynamic 1 microsecond simulations for Erlotinib, **41** (UNC-CA-353) and **45** (UNC-CA-359).


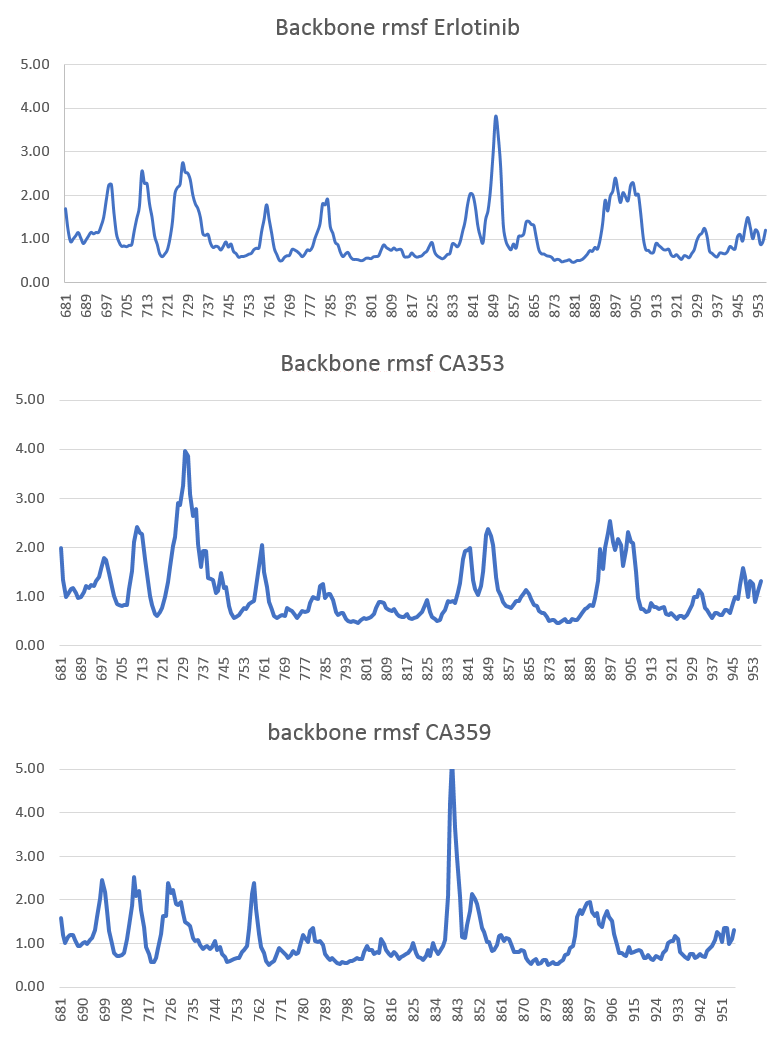


**Figure S3**. Molecular Dynamic 0-0.5 microsecond simulations for Erlotinib, **41** (UNC-CA-353) and **45** (UNC-CA-359) showing backbone interactions in the main ATP binding pocket.

**3. Kinome Scans of 41 and 45**

**Table S4**. Profiling of two compounds at two concentrations against 320 wild-type protein kinases; singlicate measurement; Residual activities (% of control).

| # | **Kinase Name** | **Kinase Family*** | **41** (UNC-CA353) | | **45** (UNC-CA359) | |
| --- | --- | --- | --- | --- | --- | --- |
|  |  |  | **Assay Conc. (M)** | | | |
|  |  |  | **1.0E-07** | **1.0E-06** | **1.0E-07** | **1.0E-06** |
| **1** | **ABL1** | **TK** | 85 | 72 | 100 | 59 |
| **2** | **ABL2** | **TK** | 106 | 97 | 96 | 68 |
| **3** | **ACK1** | **TK** | 134 | 98 | 100 | 63 |
| **4** | **ACV-R1** | **TKL** | 91 | 86 | 98 | 93 |
| **5** | **ACV-R1B** | **TKL** | 105 | 126 | 99 | 128 |
| **6** | **ACV-R2A** | **TKL** | 96 | 92 | 101 | 96 |
| **7** | **ACV-R2B** | **TKL** | 91 | 84 | 83 | 85 |
| **8** | **ACV-RL1** | **TKL** | 102 | 105 | 110 | 106 |
| **9** | **AKT1 aa106-480** | **AGC** | 96 | 94 | 100 | 101 |
| **10** | **AKT2 aa107-481** | **AGC** | 98 | 86 | 112 | 94 |
| **11** | **AKT3 aa106-479** | **AGC** | 94 | 97 | 96 | 101 |
| **12** | **ALK (GST-HIS-tag)** | **TK** | 102 | 93 | 102 | 63 |
| **13** | **AMPK-alpha1 aa1-550** | **CAMK** | 106 | 107 | 91 | 98 |
| **14** | **ARK5** | **CAMK** | 110 | 108 | 104 | 114 |
| **15** | **ASK1** | **STE** | 95 | 99 | 93 | 99 |
| **16** | **Aurora-A** | **OTHER** | 95 | 81 | 99 | 80 |
| **17** | **Aurora-B** | **OTHER** | 75 | 30 | 91 | 43 |
| **18** | **Aurora-C** | **OTHER** | 89 | 70 | 98 | 85 |
| **19** | **AXL** | **TK** | 116 | 97 | 103 | 80 |
| **20** | **BLK** | **TK** | 99 | 93 | 99 | 62 |
| **21** | **BMPR1A** | **TKL** | 109 | 96 | 102 | 110 |
| **22** | **BMX** | **TK** | 86 | 77 | 92 | 81 |
| **23** | **B-RAF** | **TKL** | 145 | 115 | 112 | 108 |
| **24** | **BRK** | **TK** | 100 | 110 | 96 | 70 |
| **25** | **BRSK1** | **CAMK** | 100 | 91 | 89 | 97 |
| **26** | **BRSK2** | **CAMK** | 98 | 100 | 106 | 101 |
| **27** | **BTK** | **TK** | 103 | 100 | 100 | 65 |
| **28** | **BUB1B** | **OTHER** | 122 | 131 | 137 | 115 |
| **29** | **CAMK1D** | **CAMK** | 86 | 77 | 99 | 84 |
| **30** | **CAMK2A** | **CAMK** | 99 | 93 | 104 | 101 |
| **31** | **CAMK2B** | **CAMK** | 84 | 86 | 81 | 103 |
| **32** | **CAMK2D** | **CAMK** | 103 | 89 | 91 | 95 |
| **33** | **CAMK2G** | **CAMK** | 118 | 93 | 116 | 91 |
| **34** | **CAMK4** | **CAMK** | 84 | 91 | 84 | 89 |
| **35** | **CAMKK1** | **OTHER** | 87 | 84 | 91 | 110 |
| **36** | **CAMKK2** | **OTHER** | 96 | 71 | 79 | 74 |
| **37** | **CDC42BPA** | **AGC** | 140 | 141 | 114 | 113 |
| **38** | **CDC42BPB** | **AGC** | 101 | 98 | 98 | 95 |
| **39** | **CDC7/DBF4** | **OTHER** | 89 | 90 | 107 | 88 |
| **40** | **CDK1/CycA2** | **CMGC** | 91 | 77 | 83 | 83 |
| **41** | **CDK1/CycB1** | **CMGC** | 84 | 79 | 89 | 78 |
| **42** | **CDK1/CycE1** | **CMGC** | 116 | 101 | 97 | 91 |
| **43** | **CDK16/CycY** | **CMGC** | 84 | 84 | 86 | 90 |
| **44** | **CDK19/CycC** | **CMGC** | 91 | 93 | 104 | 97 |
| **45** | **CDK2/CycA2** | **CMGC** | 88 | 90 | 95 | 97 |
| **46** | **CDK2/CycE1** | **CMGC** | 97 | 76 | 91 | 86 |
| **47** | **CDK3/CycC** | **CMGC** | 90 | 87 | 92 | 86 |
| **48** | **CDK3/CycE1** | **CMGC** | 115 | 96 | 117 | 102 |
| **49** | **CDK4/CycD1** | **CMGC** | 75 | 79 | 87 | 95 |
| **50** | **CDK4/CycD3** | **CMGC** | 94 | 87 | 94 | 87 |
| **51** | **CDK5/p25NCK** | **CMGC** | 119 | 100 | 98 | 103 |
| **52** | **CDK5/p35NCK** | **CMGC** | 87 | 86 | 88 | 88 |
| **53** | **CDK6/CycD1** | **CMGC** | 92 | 98 | 96 | 86 |
| **54** | **CDK6/CycD3** | **CMGC** | 102 | 103 | 95 | 105 |
| **55** | **CDK7/CycH/MAT1** | **CMGC** | 93 | 81 | 74 | 79 |
| **56** | **CDK8/CycC** | **CMGC** | 91 | 92 | 97 | 95 |
| **57** | **CDK9/CycK** | **CMGC** | 99 | 80 | 103 | 86 |
| **58** | **CDK9/CycT1** | **CMGC** | 95 | 92 | 90 | 88 |
| **59** | **CHK1** | **CAMK** | 94 | 85 | 94 | 74 |
| **60** | **CHK2** | **CAMK** | 105 | 83 | 94 | 78 |
| **61** | **CK1-alpha1** | **CK1** | 104 | 99 | 98 | 95 |
| **62** | **CK1-delta** | **CK1** | 134 | 115 | 129 | 111 |
| **63** | **CK1-epsilon** | **CK1** | 124 | 118 | 124 | 120 |
| **64** | **CK1-gamma1** | **CK1** | 125 | 123 | 112 | 100 |
| **65** | **CK1-gamma2** | **CK1** | 119 | 112 | 111 | 109 |
| **66** | **CK1-gamma3** | **CK1** | 121 | 118 | 116 | 123 |
| **67** | **CK2-alpha1** | **OTHER** | 104 | 99 | 109 | 101 |
| **68** | **CK2-alpha2** | **OTHER** | 115 | 115 | 118 | 111 |
| **69** | **CLK1** | **CMGC** | 89 | 89 | 102 | 79 |
| **70** | **CLK2** | **CMGC** | 122 | 106 | 119 | 105 |
| **71** | **CLK3** | **CMGC** | 146 | 128 | 127 | 133 |
| **72** | **CLK4** | **CMGC** | 111 | 112 | 100 | 69 |
| **73** | **COT** | **STE** | 91 | 91 | 98 | 123 |
| **74** | **CSF1-R** | **TK** | 130 | 123 | 127 | 108 |
| **75** | **CSK** | **TK** | 110 | 92 | 106 | 92 |
| **76** | **DAPK1** | **CAMK** | 125 | 112 | 126 | 116 |
| **77** | **DAPK2** | **CAMK** | 113 | 118 | 132 | 101 |
| **78** | **DAPK3** | **CAMK** | 110 | 109 | 112 | 114 |
| **79** | **DCAMKL2** | **CAMK** | 87 | 90 | 97 | 103 |
| **80** | **DDR2** | **TK** | 91 | 81 | 87 | 35 |
| **81** | **DMPK** | **AGC** | 121 | 106 | 103 | 104 |
| **82** | **DNA-PK** | **ATYPICAL** | 96 | 100 | 108 | 85 |
| **83** | **DYRK1A** | **CMGC** | 84 | 75 | 85 | 91 |
| **84** | **DYRK1B** | **CMGC** | 116 | 90 | 105 | 111 |
| **85** | **DYRK2** | **CMGC** | 84 | 61 | 89 | 71 |
| **86** | **DYRK3** | **CMGC** | 96 | 81 | 97 | 69 |
| **87** | **DYRK4** | **CMGC** | 99 | 96 | 116 | 97 |
| **88** | **EEF2K** | **ATYPICAL** | 94 | 87 | 91 | 83 |
| **89** | **EGF-R** | **TK** | 28 | 8 | 2 | 0 |
| **90** | **EIF2AK2** | **OTHER** | 72 | 58 | 77 | 77 |
| **91** | **EIF2AK3** | **OTHER** | 86 | 95 | 90 | 82 |
| **92** | **EPHA1** | **TK** | 106 | 78 | 91 | 80 |
| **93** | **EPHA2** | **TK** | 90 | 91 | 87 | 81 |
| **94** | **EPHA3** | **TK** | 118 | 119 | 128 | 111 |
| **95** | **EPHA4** | **TK** | 96 | 83 | 99 | 61 |
| **96** | **EPHA5** | **TK** | 128 | 126 | 115 | 92 |
| **97** | **EPHA6** | **TK** | 94 | 75 | 89 | 35 |
| **98** | **EPHA7** | **TK** | 85 | 94 | 84 | 58 |
| **99** | **EPHA8** | **TK** | 110 | 118 | 112 | 103 |
| **100** | **EPHB1** | **TK** | 103 | 87 | 86 | 82 |
| **101** | **EPHB2** | **TK** | 79 | 58 | 50 | 11 |
| **102** | **EPHB3** | **TK** | 110 | 100 | 99 | 113 |
| **103** | **EPHB4** | **TK** | 89 | 92 | 101 | 82 |
| **104** | **ERBB2** | **TK** | 96 | 78 | 44 | 11 |
| **105** | **ERBB4** | **TK** | 92 | 44 | 34 | 7 |
| **106** | **ERK1** | **CMGC** | 91 | 97 | 92 | 95 |
| **107** | **ERK2** | **CMGC** | 90 | 86 | 94 | 87 |
| **108** | **ERK5** | **CMGC** | 92 | 74 | 96 | 85 |
| **109** | **ERK7** | **CMGC** | 94 | 96 | 104 | 90 |
| **110** | **FAK aa2-1052** | **TK** | 138 | 106 | 109 | 95 |
| **111** | **FER** | **TK** | 113 | 110 | 115 | 93 |
| **112** | **FES** | **TK** | 118 | 93 | 107 | 97 |
| **113** | **FGF-R1** | **TK** | 107 | 85 | 100 | 92 |
| **114** | **FGF-R2** | **TK** | 88 | 85 | 87 | 69 |
| **115** | **FGF-R3** | **TK** | 99 | 93 | 79 | 70 |
| **116** | **FGF-R4** | **TK** | 96 | 88 | 100 | 96 |
| **117** | **FGR** | **TK** | 129 | 114 | 125 | 94 |
| **118** | **FLT3** | **TK** | 95 | 94 | 88 | 56 |
| **119** | **FRK** | **TK** | 93 | 106 | 96 | 74 |
| **120** | **FYN** | **TK** | 107 | 101 | 110 | 76 |
| **121** | **GRK2** | **AGC** | 104 | 117 | 96 | 111 |
| **122** | **GRK3** | **AGC** | 131 | 127 | 123 | 118 |
| **123** | **GRK4** | **AGC** | 128 | 113 | 121 | 126 |
| **124** | **GRK5** | **AGC** | 108 | 111 | 107 | 111 |
| **125** | **GRK6** | **AGC** | 124 | 120 | 133 | 101 |
| **126** | **GRK7** | **AGC** | 124 | 116 | 115 | 121 |
| **127** | **GSG2** | **OTHER** | 113 | 112 | 115 | 109 |
| **128** | **GSK3-alpha** | **CMGC** | 148 | 143 | 136 | 122 |
| **129** | **GSK3-beta** | **CMGC** | 96 | 84 | 84 | 85 |
| **130** | **HCK** | **TK** | 110 | 90 | 102 | 72 |
| **131** | **HIPK1** | **CMGC** | 98 | 87 | 79 | 80 |
| **132** | **HIPK2** | **CMGC** | 97 | 101 | 98 | 94 |
| **133** | **HIPK3** | **CMGC** | 98 | 103 | 102 | 97 |
| **134** | **HIPK4** | **CMGC** | 104 | 97 | 98 | 57 |
| **135** | **HRI** | **OTHER** | 108 | 89 | 101 | 87 |
| **136** | **IGF1-R** | **TK** | 109 | 103 | 122 | 101 |
| **137** | **IKK-alpha** | **OTHER** | 109 | 100 | 106 | 95 |
| **138** | **IKK-beta** | **OTHER** | 199 | 124 | 121 | 107 |
| **139** | **IKK-epsilon** | **OTHER** | 108 | 94 | 108 | 101 |
| **140** | **INS-R** | **TK** | 105 | 96 | 97 | 82 |
| **141** | **INSR-R** | **TK** | 96 | 93 | 103 | 99 |
| **142** | **IRAK1** | **TKL** | 109 | 103 | 109 | 105 |
| **143** | **IRAK4 (untagged)** | **TKL** | 96 | 84 | 90 | 69 |
| **144** | **ITK** | **TK** | 99 | 107 | 88 | 99 |
| **145** | **JAK1 aa583-1154 wt** | **TK** | 98 | 90 | 111 | 102 |
| **146** | **JAK2** | **TK** | 105 | 98 | 102 | 92 |
| **147** | **JAK3** | **TK** | 108 | 78 | 112 | 85 |
| **148** | **JNK1** | **CMGC** | 90 | 91 | 113 | 94 |
| **149** | **JNK2** | **CMGC** | 110 | 116 | 115 | 120 |
| **150** | **JNK3** | **CMGC** | 90 | 99 | 92 | 89 |
| **151** | **KIT** | **TK** | 128 | 121 | 124 | 105 |
| **152** | **LCK** | **TK** | 78 | 59 | 94 | 36 |
| **153** | **LIMK1** | **TKL** | 101 | 86 | 103 | 90 |
| **154** | **LIMK2** | **TKL** | 92 | 100 | 94 | 89 |
| **155** | **LRRK2** | **TKL** | 88 | 87 | 99 | 81 |
| **156** | **LTK** | **TK** | 106 | 77 | 100 | 54 |
| **157** | **LYN** | **TK** | 103 | 89 | 94 | 62 |
| **158** | **MAP3K1** | **STE** | 96 | 93 | 96 | 96 |
| **159** | **MAP3K10** | **STE** | 102 | 93 | 92 | 86 |
| **160** | **MAP3K11** | **STE** | 75 | 73 | 79 | 59 |
| **161** | **MAP3K7/MAP3K7IP1** | **STE** | 98 | 87 | 81 | 79 |
| **162** | **MAP3K9** | **STE** | 97 | 87 | 92 | 61 |
| **163** | **MAP4K2** | **STE** | 99 | 94 | 105 | 113 |
| **164** | **MAP4K4** | **STE** | 73 | 41 | 87 | 48 |
| **165** | **MAP4K5** | **STE** | 101 | 70 | 108 | 22 |
| **166** | **MAPKAPK2** | **CAMK** | 139 | 127 | 137 | 138 |
| **167** | **MAPKAPK3** | **CAMK** | 99 | 86 | 105 | 87 |
| **168** | **MAPKAPK5** | **CAMK** | 123 | 111 | 126 | 112 |
| **169** | **MARK1** | **CAMK** | 94 | 85 | 93 | 93 |
| **170** | **MARK2** | **CAMK** | 104 | 114 | 103 | 90 |
| **171** | **MARK3** | **CAMK** | 97 | 95 | 96 | 100 |
| **172** | **MARK4** | **CAMK** | 111 | 114 | 110 | 115 |
| **173** | **MATK** | **TK** | 100 | 95 | 108 | 95 |
| **174** | **MEK1** | **STE** | 109 | 104 | 105 | 109 |
| **175** | **MEK2** | **STE** | 101 | 80 | 102 | 105 |
| **176** | **MEK5** | **STE** | 103 | 100 | 107 | 108 |
| **177** | **MEKK2** | **STE** | 112 | 93 | 120 | 107 |
| **178** | **MEKK3** | **STE** | 106 | 107 | 115 | 117 |
| **179** | **MELK** | **CAMK** | 101 | 92 | 109 | 101 |
| **180** | **MERTK** | **TK** | 103 | 102 | 97 | 83 |
| **181** | **MET** | **TK** | 93 | 89 | 99 | 70 |
| **182** | **MINK1** | **STE** | 64 | 54 | 86 | 29 |
| **183** | **MKK4** | **STE** | 87 | 93 | 88 | 96 |
| **184** | **MKK6 S207D/T211D**** | **STE** | 90 | 98 | 95 | 90 |
| **185** | **MKK7** | **STE** | 86 | 120 | 112 | 98 |
| **186** | **MKNK1** | **CAMK** | 79 | 63 | 63 | 30 |
| **187** | **MKNK2** | **CAMK** | 92 | 74 | 78 | 46 |
| **188** | **MLK4** | **TKL** | 79 | 85 | 85 | 83 |
| **189** | **MST1** | **STE** | 80 | 84 | 89 | 83 |
| **190** | **MST2** | **STE** | 113 | 102 | 103 | 102 |
| **191** | **MST3** | **STE** | 116 | 104 | 93 | 94 |
| **192** | **MST4** | **STE** | 101 | 89 | 92 | 97 |
| **193** | **mTOR** | **ATYPICAL** | 97 | 84 | 106 | 91 |
| **194** | **MUSK** | **TK** | 94 | 96 | 94 | 87 |
| **195** | **MYLK** | **CAMK** | 98 | 58 | 92 | 71 |
| **196** | **MYLK2** | **CAMK** | 90 | 75 | 89 | 70 |
| **197** | **MYLK3** | **CAMK** | 104 | 93 | 100 | 86 |
| **198** | **NEK1** | **OTHER** | 86 | 82 | 89 | 89 |
| **199** | **NEK11** | **OTHER** | 82 | 95 | 106 | 103 |
| **200** | **NEK2** | **OTHER** | 96 | 82 | 92 | 86 |
| **201** | **NEK3** | **OTHER** | 110 | 103 | 101 | 113 |
| **202** | **NEK4** | **OTHER** | 100 | 94 | 106 | 84 |
| **203** | **NEK6** | **OTHER** | 84 | 92 | 90 | 92 |
| **204** | **NEK7** | **OTHER** | 129 | 101 | 117 | 107 |
| **205** | **NEK9** | **OTHER** | 134 | 115 | 111 | 91 |
| **206** | **NIK** | **STE** | 126 | 120 | 127 | 122 |
| **207** | **NLK** | **CMGC** | 76 | 63 | 83 | 31 |
| **208** | **p38-alpha** | **CMGC** | 103 | 71 | 96 | 56 |
| **209** | **p38-beta** | **CMGC** | 82 | 84 | 90 | 87 |
| **210** | **p38-delta** | **CMGC** | 112 | 94 | 111 | 94 |
| **211** | **p38-gamma** | **CMGC** | 87 | 78 | 90 | 85 |
| **212** | **PAK1** | **STE** | 87 | 92 | 91 | 95 |
| **213** | **PAK2** | **STE** | 108 | 102 | 101 | 104 |
| **214** | **PAK3** | **STE** | 96 | 75 | 98 | 92 |
| **215** | **PAK4** | **STE** | 103 | 82 | 103 | 95 |
| **216** | **PAK6** | **STE** | 97 | 88 | 98 | 87 |
| **217** | **PAK7** | **STE** | 108 | 81 | 99 | 91 |
| **218** | **PASK** | **CAMK** | 102 | 101 | 98 | 93 |
| **219** | **PBK** | **OTHER** | 98 | 110 | 91 | 80 |
| **220** | **PDGFR-alpha** | **TK** | 118 | 117 | 105 | 95 |
| **221** | **PDGFR-beta** | **TK** | 114 | 98 | 107 | 95 |
| **222** | **PDK1** | **AGC** | 96 | 77 | 93 | 79 |
| **223** | **PHKG1** | **CAMK** | 97 | 94 | 108 | 85 |
| **224** | **PHKG2** | **CAMK** | 94 | 98 | 95 | 103 |
| **225** | **PIM1** | **CAMK** | 85 | 71 | 73 | 38 |
| **226** | **PIM2** | **CAMK** | 112 | 101 | 89 | 100 |
| **227** | **PIM3** | **CAMK** | 81 | 83 | 81 | 53 |
| **228** | **PKA** | **AGC** | 88 | 82 | 93 | 90 |
| **229** | **PKC-alpha** | **AGC** | 86 | 95 | 111 | 94 |
| **230** | **PKC-beta1** | **AGC** | 110 | 96 | 95 | 88 |
| **231** | **PKC-beta2** | **AGC** | 98 | 95 | 97 | 95 |
| **232** | **PKC-delta** | **AGC** | 92 | 88 | 115 | 89 |
| **233** | **PKC-epsilon** | **AGC** | 82 | 89 | 89 | 88 |
| **234** | **PKC-eta** | **AGC** | 93 | 91 | 90 | 75 |
| **235** | **PKC-gamma** | **AGC** | 99 | 89 | 87 | 88 |
| **236** | **PKC-iota** | **AGC** | 119 | 104 | 139 | 96 |
| **237** | **PKC-mu** | **AGC** | 111 | 89 | 92 | 73 |
| **238** | **PKC-nu** | **AGC** | 87 | 86 | 99 | 84 |
| **239** | **PKC-theta** | **AGC** | 101 | 102 | 109 | 101 |
| **240** | **PKC-zeta** | **AGC** | 87 | 76 | 73 | 85 |
| **241** | **PKMYT1** | **OTHER** | 88 | 79 | 114 | 95 |
| **242** | **PLK1** | **OTHER** | 98 | 98 | 91 | 77 |
| **243** | **PLK3** | **OTHER** | 109 | 102 | 103 | 98 |
| **244** | **PRK1** | **AGC** | 96 | 96 | 91 | 91 |
| **245** | **PRK2** | **AGC** | 85 | 88 | 92 | 95 |
| **246** | **PRKD2** | **CAMK** | 97 | 84 | 80 | 73 |
| **247** | **PRKG1** | **AGC** | 88 | 72 | 106 | 101 |
| **248** | **PRKG2** | **AGC** | 100 | 20 | 118 | 80 |
| **249** | **PRKX** | **AGC** | 138 | 123 | 117 | 118 |
| **250** | **PYK2** | **TK** | 103 | 97 | 95 | 92 |
| **251** | **RAF1 Y340D/Y341D (untagged)**** | **TKL** | 95 | 99 | 83 | 88 |
| **252** | **RET** | **TK** | 102 | 85 | 101 | 51 |
| **253** | **RIPK2** | **TKL** | 107 | 96 | 59 | 13 |
| **254** | **RIPK5** | **TKL** | 96 | 85 | 84 | 71 |
| **255** | **ROCK1** | **AGC** | 84 | 91 | 98 | 83 |
| **256** | **ROCK2** | **AGC** | 100 | 88 | 90 | 84 |
| **257** | **RON** | **TK** | 77 | 63 | 90 | 82 |
| **258** | **ROS** | **TK** | 95 | 88 | 96 | 89 |
| **259** | **RPS6KA1** | **AGC** | 103 | 99 | 88 | 74 |
| **260** | **RPS6KA2** | **AGC** | 97 | 94 | 100 | 86 |
| **261** | **RPS6KA3** | **AGC** | 99 | 96 | 119 | 115 |
| **262** | **RPS6KA4** | **AGC** | 95 | 93 | 95 | 83 |
| **263** | **RPS6KA5** | **AGC** | 112 | 99 | 104 | 107 |
| **264** | **RPS6KA6** | **AGC** | 103 | 94 | 93 | 70 |
| **265** | **S6K** | **AGC** | 135 | 123 | 122 | 121 |
| **266** | **S6K-beta** | **AGC** | 96 | 102 | 105 | 86 |
| **267** | **SAK** | **OTHER** | 95 | 77 | 99 | 82 |
| **268** | **SGK1** | **AGC** | 103 | 77 | 99 | 88 |
| **269** | **SGK2** | **AGC** | 101 | 86 | 94 | 98 |
| **270** | **SGK3** | **AGC** | 91 | 86 | 93 | 89 |
| **271** | **SIK1** | **CAMK** | 93 | 77 | 101 | 87 |
| **272** | **SIK2** | **CAMK** | 92 | 83 | 97 | 67 |
| **273** | **SIK3** | **CAMK** | 77 | 72 | 83 | 58 |
| **274** | **SLK** | **STE** | 67 | 32 | 76 | 18 |
| **275** | **SNARK** | **CAMK** | 101 | 83 | 88 | 87 |
| **276** | **SNK** | **OTHER** | 97 | 89 | 99 | 87 |
| **277** | **SRC (GST-HIS-tag)** | **TK** | 108 | 86 | 100 | 90 |
| **278** | **SRMS** | **TK** | 113 | 98 | 124 | 103 |
| **279** | **SRPK1** | **CMGC** | 96 | 89 | 100 | 97 |
| **280** | **SRPK2** | **CMGC** | 131 | 115 | 115 | 151 |
| **281** | **STK17A** | **CAMK** | 104 | 103 | 102 | 83 |
| **282** | **STK23** | **CAMK** | 93 | 94 | 97 | 87 |
| **283** | **STK25** | **STE** | 95 | 100 | 111 | 104 |
| **284** | **STK33** | **CAMK** | 98 | 89 | 100 | 76 |
| **285** | **STK39** | **STE** | 106 | 98 | 102 | 91 |
| **286** | **SYK aa1-635** | **TK** | 94 | 94 | 105 | 104 |
| **287** | **TAOK2** | **STE** | 110 | 96 | 109 | 106 |
| **288** | **TAOK3** | **STE** | 87 | 93 | 92 | 106 |
| **289** | **TBK1** | **OTHER** | 106 | 98 | 106 | 89 |
| **290** | **TEC** | **TK** | 96 | 90 | 108 | 92 |
| **291** | **TGFB-R1** | **TKL** | 86 | 81 | 84 | 75 |
| **292** | **TGFB-R2** | **TKL** | 106 | 87 | 108 | 83 |
| **293** | **TIE2** | **TK** | 84 | 87 | 111 | 82 |
| **294** | **TLK1** | **AGC** | 110 | 100 | 112 | 108 |
| **295** | **TLK2** | **AGC** | 117 | 109 | 111 | 103 |
| **296** | **TNK1** | **TK** | 130 | 112 | 117 | 83 |
| **297** | **TRK-A** | **TK** | 100 | 107 | 106 | 87 |
| **298** | **TRK-B** | **TK** | 107 | 98 | 98 | 97 |
| **299** | **TRK-C** | **TK** | 115 | 105 | 136 | 113 |
| **300** | **TSF1** | **OTHER** | 93 | 94 | 95 | 104 |
| **301** | **TSK2** | **CAMK** | 113 | 123 | 109 | 331 |
| **302** | **TSSK1** | **CAMK** | 142 | 105 | 127 | 111 |
| **303** | **TTBK1** | **CK1** | 113 | 102 | 109 | 101 |
| **304** | **TTBK2** | **CK1** | 102 | 101 | 96 | 95 |
| **305** | **TTK** | **OTHER** | 100 | 81 | 97 | 83 |
| **306** | **TXK** | **TK** | 90 | 79 | 91 | 52 |
| **307** | **TYK2** | **TK** | 101 | 94 | 96 | 114 |
| **308** | **TYRO3** | **TK** | 100 | 91 | 93 | 58 |
| **309** | **VEGF-R1** | **TK** | 92 | 113 | 119 | 85 |
| **310** | **VEGF-R2** | **TK** | 96 | 74 | 81 | 25 |
| **311** | **VEGF-R3** | **TK** | 121 | 119 | 120 | 112 |
| **312** | **VRK1** | **CK1** | 109 | 98 | 104 | 93 |
| **313** | **VRK2** | **CK1** | 95 | 97 | 87 | 232 |
| **314** | **WEE1** | **OTHER** | 112 | 87 | 109 | 76 |
| **315** | **WNK1** | **OTHER** | 84 | 70 | 93 | 95 |
| **316** | **WNK2** | **OTHER** | 108 | 82 | 97 | 92 |
| **317** | **WNK3** | **OTHER** | 118 | 115 | 124 | 105 |
| **318** | **YES** | **TK** | 101 | 83 | 100 | 77 |
| **319** | **ZAK** | **TKL** | 98 | 85 | 100 | 80 |
| **320** | **ZAP70** | **TK** | 111 | 103 | 100 | 104 |

*Classification of protein kinase families (Manning et al. *Science*, **2002**, *298*, 5600, 1912-1934):

AGC: containing PKA, PKG and PKC families

CAMK: Calcium/Calmoduline-dependent protein kinases

CK1: Casein kinase 1 -like

CMGC: containing CDK, MAPK ,GSK3 and CLK families

TK: Tyrosine Kinase

TKL: Tyrosine Kinase-like

STE: Homologs of Yeast Sterile 7, Sterile 11, Sterile 20 Kinases

** Constitutively active kinase

**4. In-Cell EGFR Dose Curves**
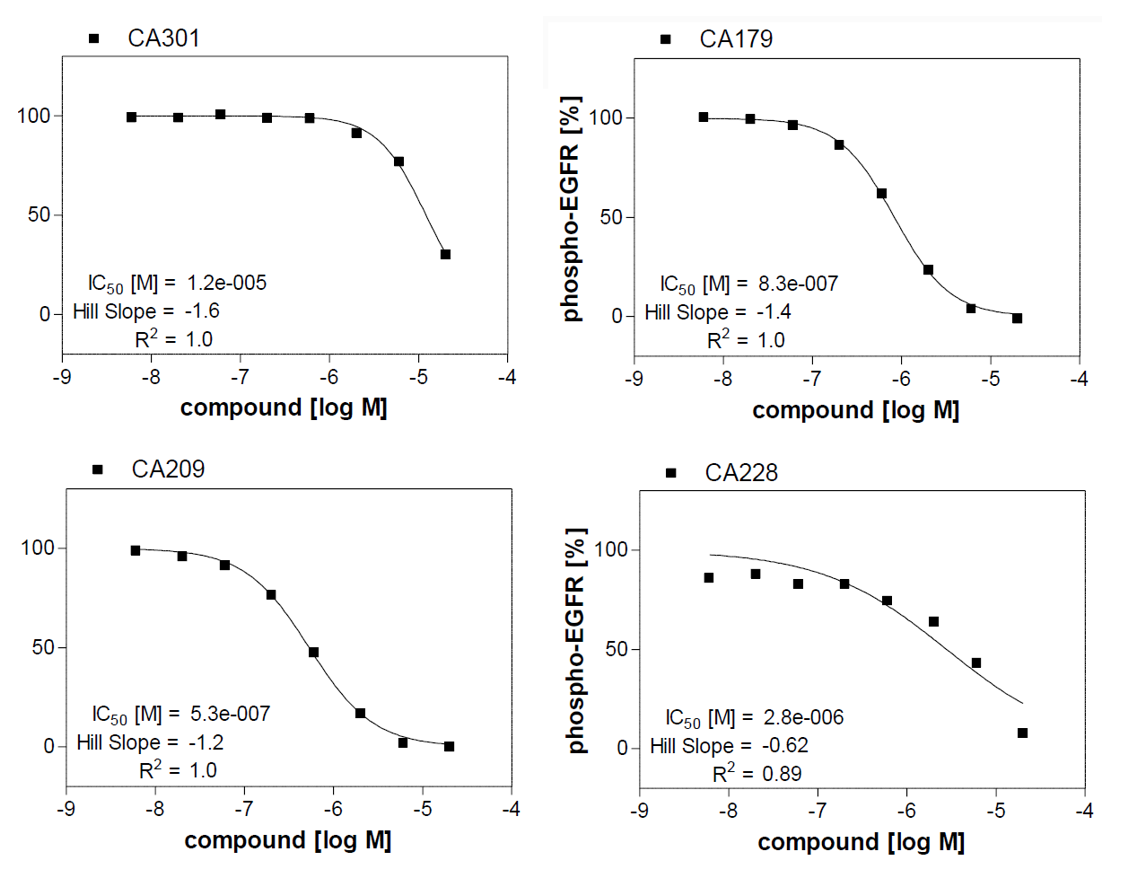


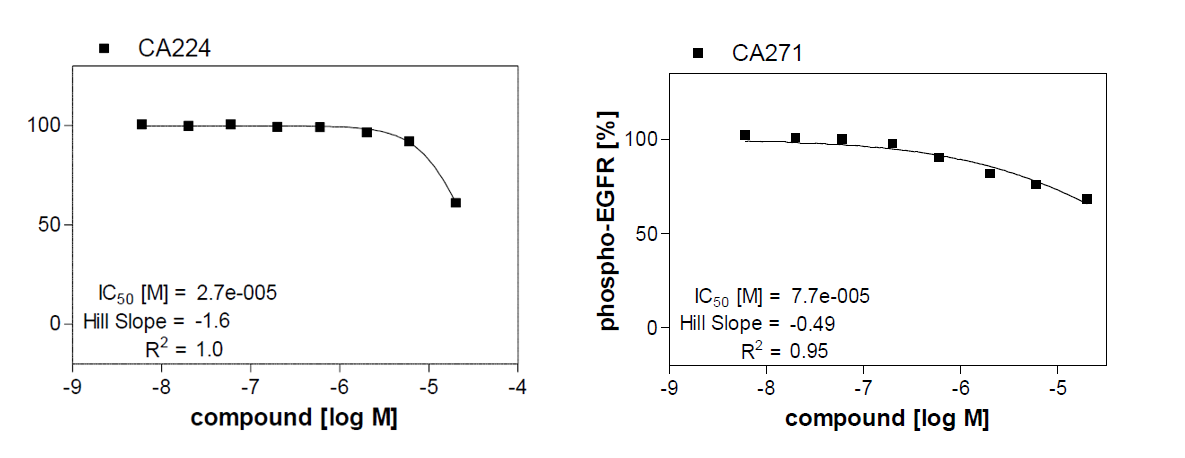


**Figure S4**. In-Cell EGFR Dose Curves: **6** (CA301), **9** (CA179), **11** (CA209), **15** (CA228), **16** (CA224) and **17** (CA271).


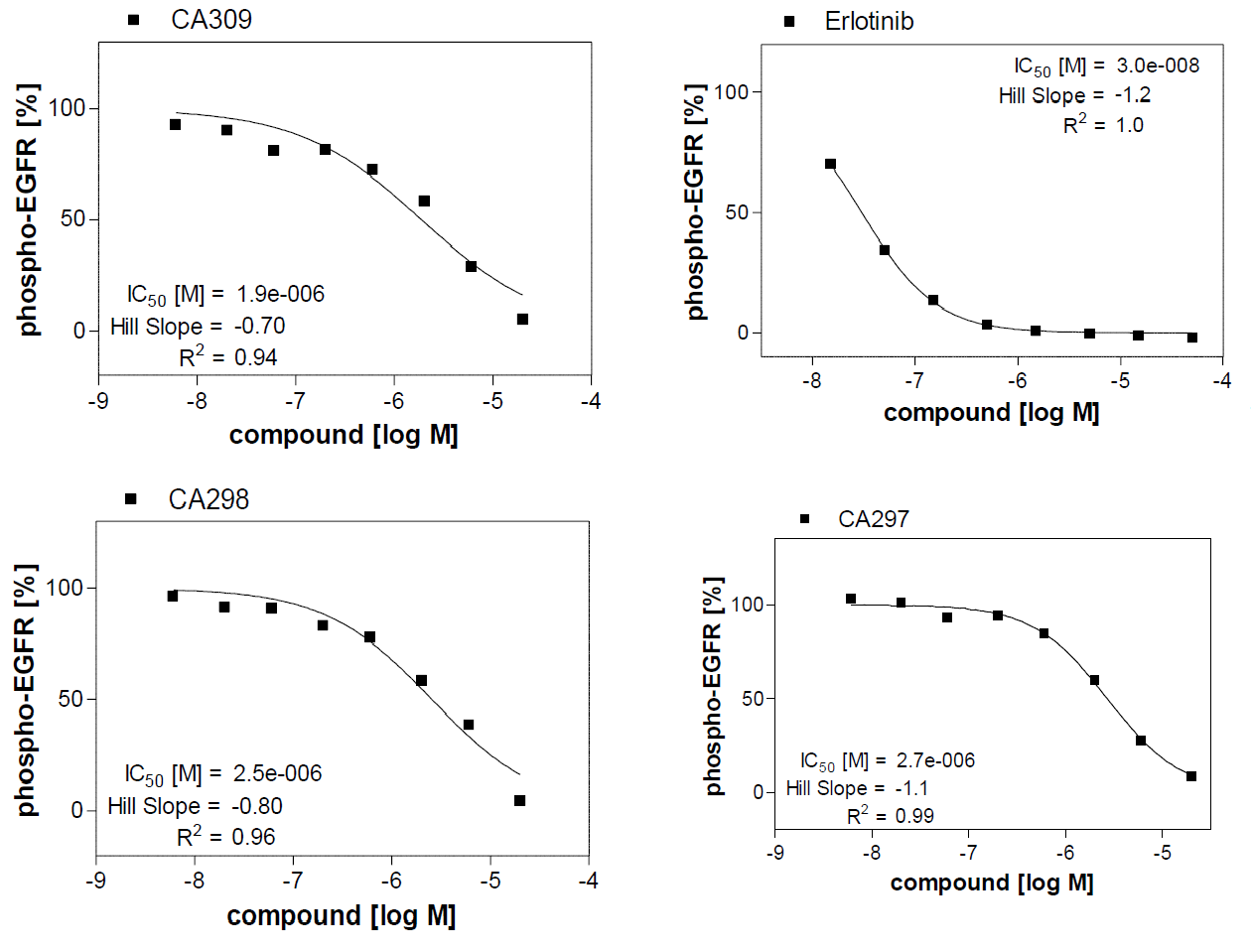


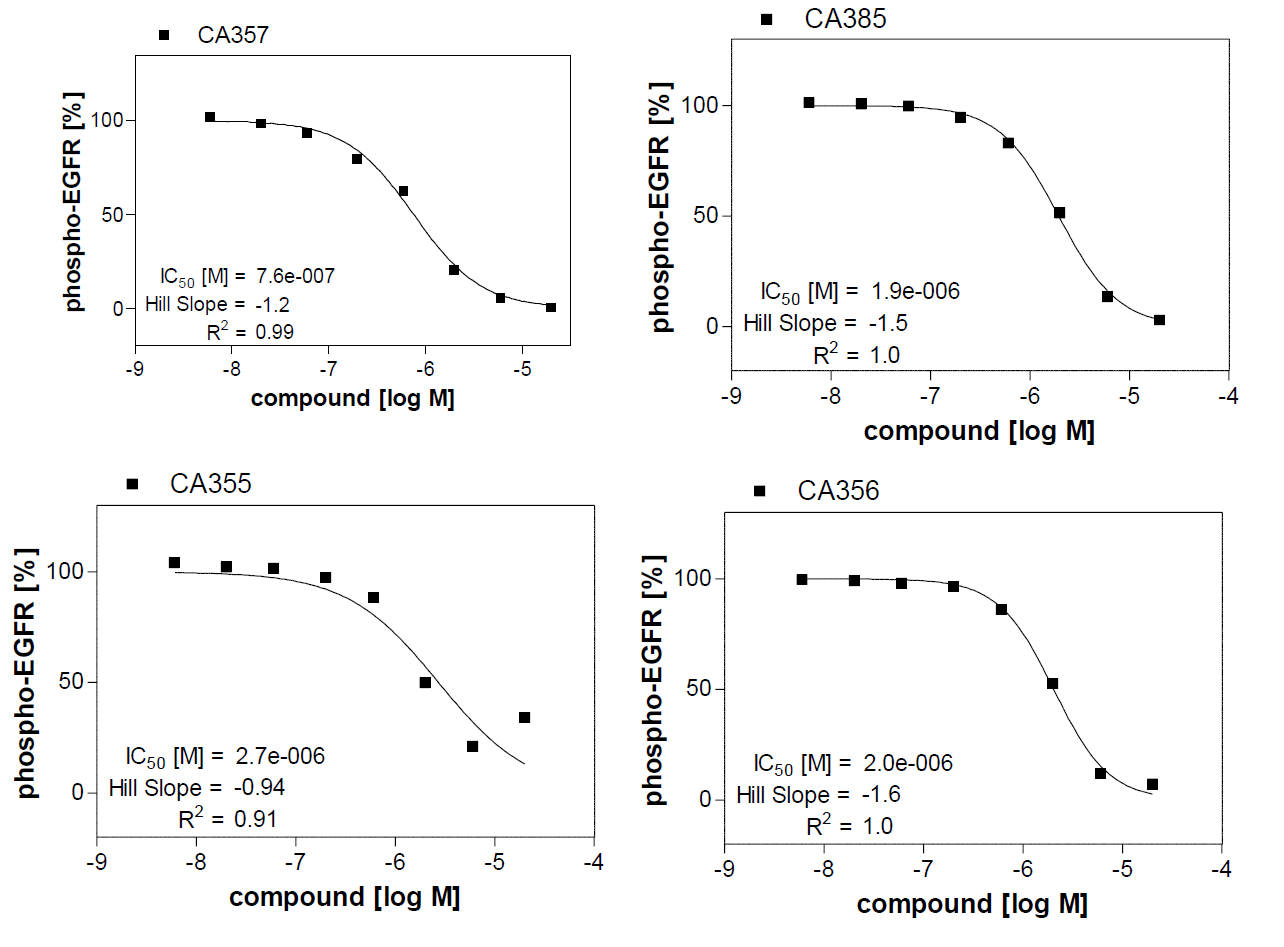


**Figure S5**. In-Cell EGFR Dose Curves: **21** (CA309), Erlotinib, **28** (CA298), **32** (CA297), **34** (CA357), **35** (CA385), **36** (CA355) and **37** (CA356).


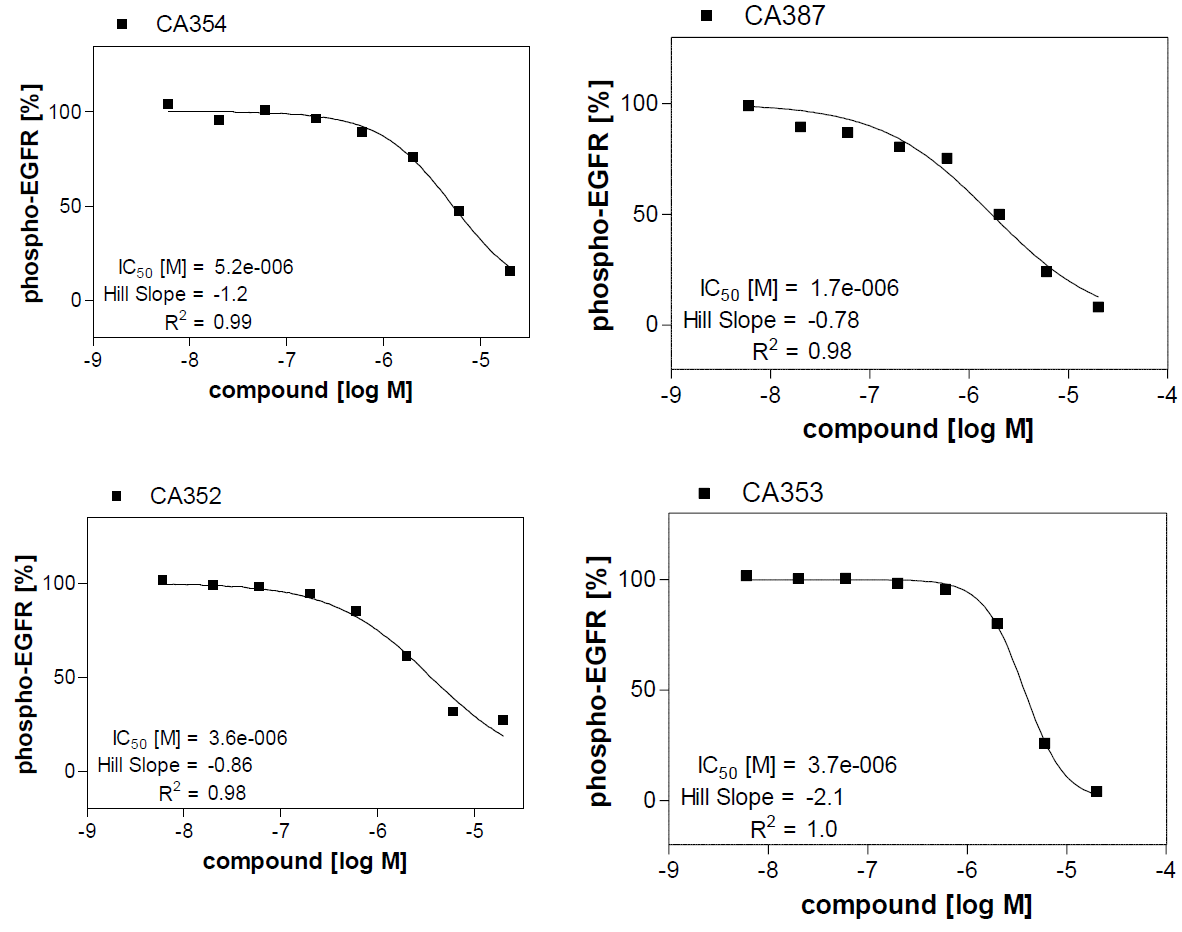


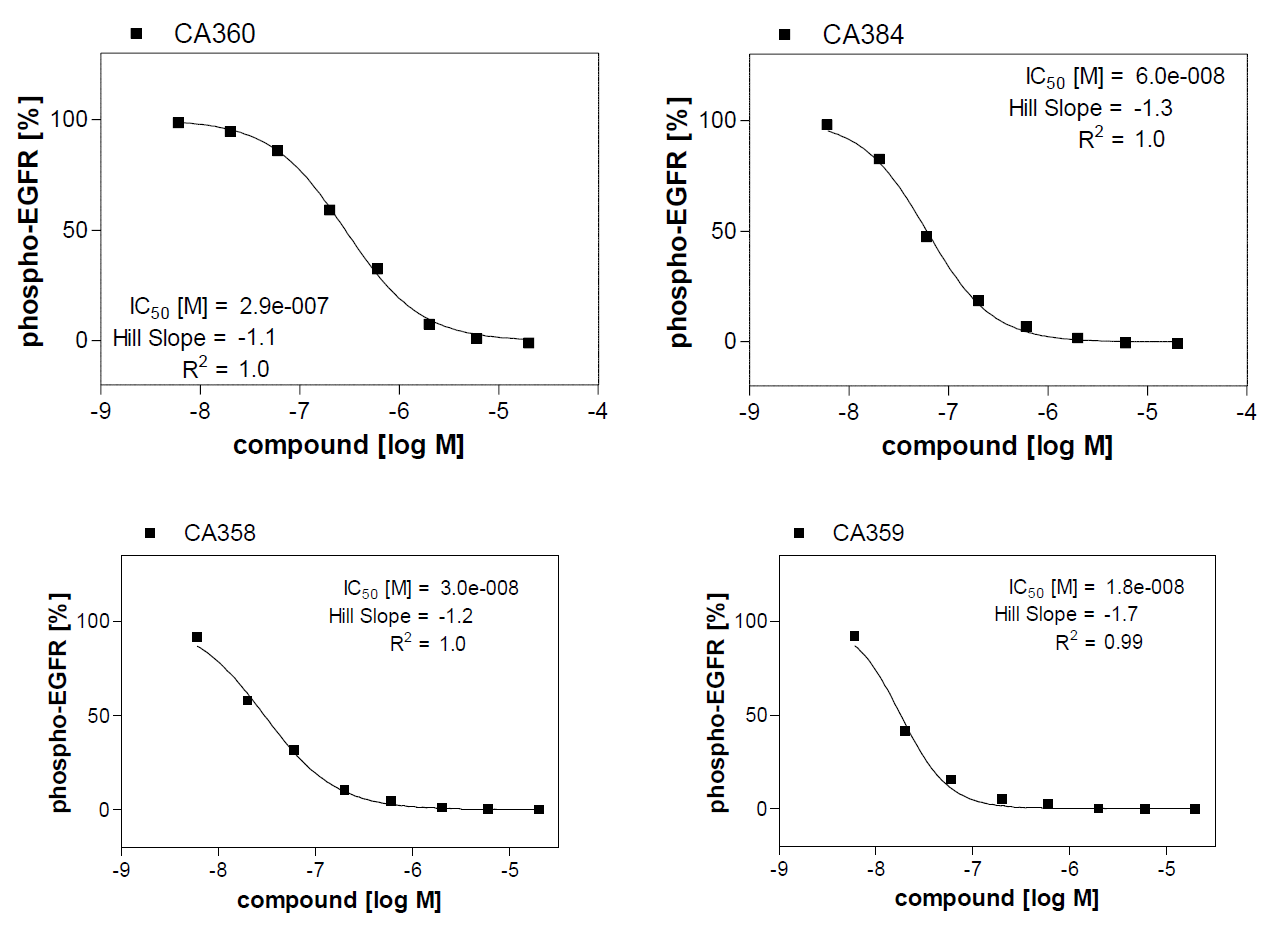


**Figure S6**. In-Cell EGFR Dose Curves: **38** (CA354), **39** (CA387), **40** (CA352), **41** (UNC-CA353), **42** (CA360), 43 (CA384), **44** (CA358), **45** (UNC-CA359).

**5. Labbook Numbers and SMILES.**

**Table S5.** Paper ID, Labbook codes and SMILES of compounds included in the manuscript.

| **Paper ID** | **Labbook** | **SMILES** |
| --- | --- | --- |
|  | **Code** |  |
| **1** | **CA176** | COC1=CC2=C(NC3=CC=CC(C#C)=C3)N=CN=C2C=C1OC |
| **2** | **CA204** | C#CC1=CC(NC2=C3C(C=CC(OC)=C3)=NC=N2)=CC=C1 |
| **3** | **CA303** | C#CC1=CC(NC2=C3C(C=CC=C3)=NC=N2)=CC=C1 |
| **4** | **CA214** | C#CC1=CC(NC2=C3C(C=CC(C)=C3)=NC=N2)=CC=C1 |
| **5** | **CA192** | FC1=CC2=C(NC3=CC=CC(C#C)=C3)N=CN=C2C=C1 |
| **6** | **CA301** | FC1=CC2=C(NC3=CC=CC(C#C)=C3)N=CN=C2C=C1F |
| **7** | **CA200** | ClC1=CC2=C(NC3=CC=CC(C#C)=C3)N=CN=C2C=C1 |
| **8** | **CA185** | BrC1=CC2=C(NC3=CC=CC(C#C)=C3)N=CN=C2C=C1 |
| **9** | **CA179** | IC1=CC2=C(NC3=CC=CC(C#C)=C3)N=CN=C2C=C1 |
| **10** | **CA240** | C#CC1=CC(NC2=C3C(C=CC(C(F)(F)F)=C3)=NC=N2)=CC=C1 |
| **11** | **CA209** | C#CC1=CC(NC2=C3C(C=C(OC)C=C3)=NC=N2)=CC=C1 |
| **12** | **CA219** | FC1=CC2=NC=NC(NC3=CC=CC(C#C)=C3)=C2C=C1 |
| **13** | **CA197** | ClC1=CC2=NC=NC(NC3=CC=CC(C#C)=C3)=C2C=C1.C |
| **14** | **CA211** | BrC1=CC2=NC=NC(NC3=CC=CC(C#C)=C3)=C2C=C1 |
| **15** | **CA228** | IC1=CC2=NC=NC(NC3=CC=CC(C#C)=C3)=C2C=C1 |
| **16** | **CA224** | C#CC1=CC(NC2=C3C(C=C(C(F)(F)F)C=C3)=NC=N2)=CC=C1 |
| **17** | **CA271** | C#CC1=CC(NC2=C3C(C=C(C#N)C=C3)=NC=N2)=CC=C1 |
| **18** | **CA207** | C#CC1=CC(NC2=C3C(C=CC(C#N)=C3)=NC=N2)=CC=C1 |
| **19** | **CA304** | C#CC1=CC(NC2=C3C(C=CC(S(=O)(C)=O)=C3)=NC=N2)=CC=C1 |
| **20** | **CA310** | C#CC1=CC(NC2=C3C=C(OCO4)C4=CC3=NC=N2)=CC=C1 |
| **21** | **CA309** | C#CC1=CC(NC2=C3C=C(OCCO4)C4=CC3=NC=N2)=CC=C1 |
| **22** | **CA252** | C#CC1=CC(NC2=C3C(C=C(OC)C(OC)=C3)=NC=C2C#N)=CC=C1 |
| **23** | **CA251** | C#CC1=CC(NC2=C3C(C=CC(OC)=C3)=NC=C2C#N)=CC=C1 |
| **24** | **CA250** | C#CC1=CC(NC2=C3C(C=CC=C3)=NC=C2C#N)=CC=C1 |
| **25** | **CA272** | FC1=CC2=C(NC3=CC=CC(C#C)=C3)C(C#N)=CN=C2C=C1 |
| **26** | **CA273** | ClC1=CC=C2N=CC(C#N)=C(NC3=CC(C#C)=CC=C3)C2=C1 |
| **27** | **CA282** | BrC1=CC2=C(NC3=CC=CC(C#C)=C3)C(C#N)=CN=C2C=C1 |
| **28** | **CA298** | IC1=CC2=C(NC3=CC=CC(C#C)=C3)C(C#N)=CN=C2C=C1 |
| **29** | **CA308** | C#CC1=CC(NC2=C3C(C=CC(S(=O)(C)=O)=C3)=NC=C2C#N)=CC=C1 |
| **30** | **CA300** | ClC1=CC2=NC=C(C#N)C(NC3=CC=CC(C#C)=C3)=C2C=C1 |
| **31** | **CA281** | BrC1=CC2=NC=C(C#N)C(NC3=CC=CC(C#C)=C3)=C2C=C1 |
| **32** | **CA297** | IC1=CC2=NC=C(C#N)C(NC3=CC=CC(C#C)=C3)=C2C=C1 |
| **33** | **CA287** | C#CC1=CC(NC2=C3C(C=C(OC)C=C3)=NC=C2C#N)=CC=C1 |
| **34** | **CA357** | IC1=CC2=C(NC3=C(F)C=CC(C#C)=C3)N=CN=C2C=C1 |
| **35** | **CA385** | FC(C(C#C)=CC=C1)=C1NC2=C3C(C=CC(I)=C3)=NC=C2 |
| **36** | **CA355** | IC1=CC2=C(NC3=CC=C(F)C(C#C)=C3)N=CN=C2C=C1 |
| **37** | **CA356** | IC1=CC2=C(NC3=CC=C(Cl)C(C#C)=C3)N=CN=C2C=C1 |
| **38** | **CA354** | IC1=CC2=NC=NC(NC3=CC(C#C)=CC=C3F)=C2C=C1 |
| **39** | **CA387** | C#CC1=CC=CC(NC2=C3C(C=C(I)C=C3)=NC=C2)=C1F |
| **40** | **CA352** | IC1=CC2=NC=NC(NC3=CC=C(F)C(C#C)=C3)=C2C=C1 |
| **41** | **CA353** | IC1=CC2=NC=NC(NC3=CC=C(Cl)C(C#C)=C3)=C2C=C1 |
| **42** | **CA360** | C#CC1=CC(NC2=C3C=C(OC)C(OC)=CC3=NC=N2)=C(F)C=C1 |
| **43** | **CA384** | C#CC1=CC=CC(NC2=C3C(C=C(OC)C(OC)=C3)=NC=N2)=C1F |
| **44** | **CA358** | FC1=CC=C(NC2=C3C=C(OC)C(OC)=CC3=NC=N2)C=C1C#C |
| **45** | **CA359** | ClC1=CC=C(NC2=C3C=C(OC)C(OC)=CC3=NC=N2)C=C1C#C |

**6. Mass Spectrometry Method.**

Samples were analyzed with a ThermoFisher Q Exactive HF-X (ThermoFisher, Bremen, Germany) mass spectrometer coupled with a Waters Acquity H-class liquid chromatograph system. Samples were introduced via a heated electrospray source (HESI) at a flow rate of 0.6 mL/min. Electrospray source conditions were set as: spray voltage 3.0 kV, sheath gas (nitrogen) 60 arb, auxillary gas (nitrogen) 20 arb, sweep gas (nitrogen) 0 arb, nebulizer temperature 375 degrees C, capillary temperature 380 degrees C, RF funnel 45 V. The mass range was set to 150-2000 m/z. All measurements were recorded at a resolution setting of 120,000.

Separations were conducted on a Waters Acquity UPLC BEH C18 column (2.1 x 50 mM, 1.7 uM particle size). LC conditions were set at 100 % water with 0.1 % formic acid (A) ramped linearly over 9.8 mins to 95 % acetonitrile with 0.1 % formic acid (B) and held until 10.2 mins. At 10.21 mins the gradient was switched back to 100% A and allowed to re-equilibrate until 11.25 mins. Injection volume for all samples was 3 uL.

Xcalibur (ThermoFisher, Breman, Germany) was used to analyze the data. Solutions were analyzed at 0.1 mg/mL or less based on responsiveness to the ESI mechanism. Molecular formula assignments were determined with Molecular Formula Calculator (v 1.2.3). All observed species were singly charged, as verified by unit *m/z* separation between mass spectral peaks corresponding to the ^12^C and ^13^C^12^C_c-1_ isotope for each elemental composition.
